# AMPK-PDZD8-GLS1 axis mediates calorie restriction-induced lifespan extension

**DOI:** 10.1038/s41422-024-01021-3

**Published:** 2024-09-19

**Authors:** Mengqi Li, Yu Wang, Xiaoyan Wei, Wei-Feng Cai, Yan-Hui Liu, Jianfeng Wu, Yan Chen, Jinye Xiong, Li-Feng Cui, Mingxia Zhu, Cixiong Zhang, Liyun Lin, Yong Yu, Hai-Long Piao, Sheng-Cai Lin, Chen-Song Zhang

**Affiliations:** 1 State Key Laboratory for Cellular Stress Biology, School of Life Sciences, Xiamen University, Xiamen, Fujian China; 2 Xiamen Key Laboratory of Radiation Oncology, Xiamen Cancer Center, The First Affiliated Hospital of Xiamen University, School of Medicine, Xiamen University, Xiamen, Fujian China; 3 Laboratory Animal Research Centre, Xiamen University, Xiamen, Fujian China; 4 CAS Key Laboratory of Separation Science for Analytical Chemistry, Dalian Institute of Chemical Physics, Chinese Academy of Sciences, Dalian, Liaoning China; 5 The Zhongzhou Laboratory for Integrative Biology, Henan University, Zhengzhou, Henan China

**Keywords:** Ageing, Stress signalling, Nutrient signalling

Dear Editor,

Calorie restriction (CR) that chronically reduces average daily caloric intake without causing malnutrition has been recognized as a non-pharmacological dietary behavior for improving health.^1^ The benefits of CR have been observed in various species, including yeast, nematodes, mice, and primates, showing a general link between reduced food intake and longevity.^2^ During CR, organisms undergo metabolic changes or adaptations, such as a shift from primarily consuming glucose to fatty acid and amino acid catabolism.^3,4^ However, the mechanism behind these adaptations and their relationship to the lifespan extension mediated by CR remains largely unknown.

The AMP-activated protein kinase (AMPK), a master metabolic regulator highly conserved across eukaryotes, is crucial in controlling metabolic adaptations during fasting and CR. Studies have shown that AMPK is activated under CR,^5^ and is necessary for the beneficial impacts of this dietary regimen.^6^ In addition, we previously discovered that under fasting, AMPK can promote the catabolism of glutamine through phosphorylating PDZ domain containing 8 (PDZD8) protein, which interacts with and activates glutaminase 1 (GLS1), the rate-limiting enzyme for glutaminolysis.^7^ To investigate the potential roles of the PDZD8-enhanced glutaminolysis in lifespan and healthspan extension, we utilized *Caenorhabditis elegans* as a model system. Similar to mammalian PDZD8, the PDZD8 homolog in *C*. *elegans*, known as *pdzd*-*8* (also named C53B4.4), can also be phosphorylated by AMPK when expressed in HEK293T cells (Fig. 1a). We also found that S536 in nematode pdzd-8 is the site for phosphorylation by AMPK (Supplementary information, Fig. S1a–c). Activation of nematode AMPK by treating with the nonmetabolizable glucose analog 2-deoxy-glucose (2-DG) to mimic low glucose or fasting (Supplementary information, Fig. S1d; see also ref.^8^), also promotes glutaminolysis as evidenced by the isotopic labeling experiments (Fig. 1b; Supplementary information, Fig. S1e). Re-introduction of pdzd-8, but not the AMPK-unphosphorylable mutant S536A of pdzd-8, into *PDZD8*^–/–^ MEFs, restored the low glucose-induced glutaminolysis (Supplementary information, Fig. S1f; see also validation data in this figure). Similar effects were observed with *pdzd*-*8*^–/–^ nematodes re-introduced with human wild-type (WT) PDZD8 and the AMPK-unphosphorylable mutant PDZD8-T527A (Fig. 1b; Supplementary information, Fig. S1e; see validation data in Supplementary information, Fig. S1g, h). In addition, re-introduction of the phospho-mimetic PDZD8-T527E mutant into *pdzd*-*8*^–/–^ nematodes promoted glutaminolysis regardless of 2-DG treatment (Supplementary information, Fig. S1i; see also validation data in Supplementary information, Fig. S1g, h). In line with the results from the isotopic labeling experiments, we observed pdzd-8-S536 and PDZD8-T527 phosphorylation-dependent increase of oxygen consumption rates (OCR) in MEFs under low glucose conditions and in the worms treated with 2-DG (Fig. 1c). The results above indicate that the AMPK-PDZD8-GLS1 axis is conserved in *C*. *elegans*.

**Fig. 1:**
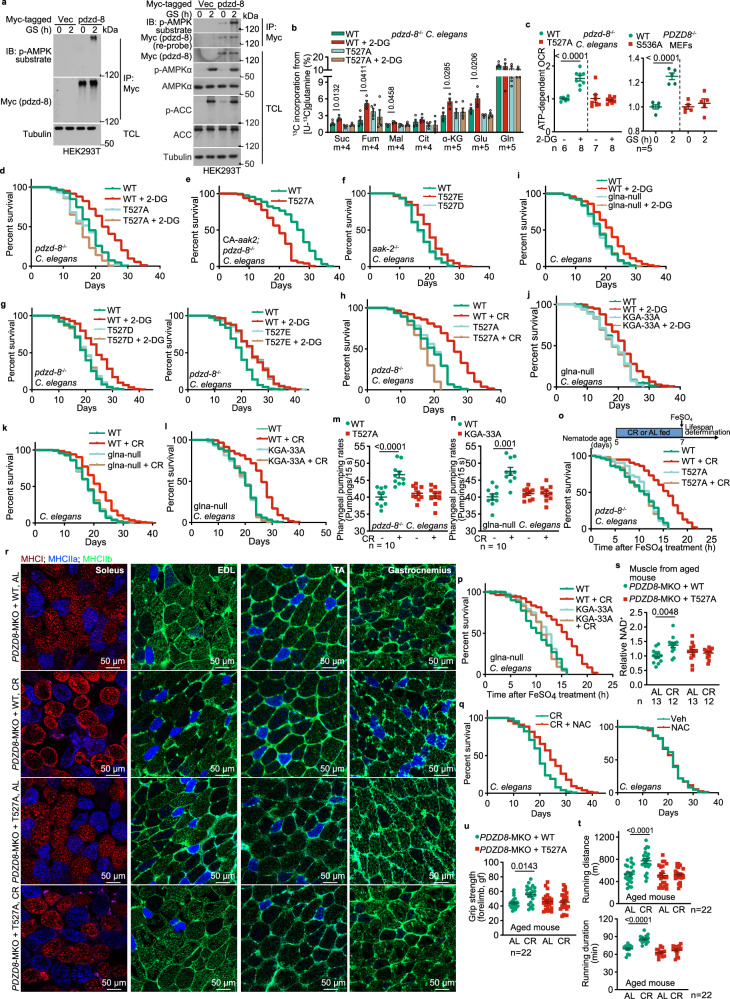
The AMPK-PDZD8-GLS1 axis mediates the rejuvenating effects of CR. **a** The nematode homolog of PDZD8 (pdzd-8) is phosphorylated by AMPK. HEK293T cells were transfected with Myc-tagged-pdzd-8 and were starved for glucose (GS) for 2 h. Cells were then lysed, and the Myc-tagged-pdzd-8 was immunoprecipitated by Myc-tag antibody, followed by immunoblotting using the pan-phospho-AMPK-substrates antibody and Myc-tag antibody on separate PVDF membranes (left panel), or the same PVDF membrane by re-probing (right panel). TCL, total cell lysate. **b**, **c** PDZD8 promotes glutaminolysis and OCR in both nematodes and mammalian cells in low glucose. The human PDZD8, or its AMPK-unphosphorylable mutant (PDZD8-T527A), was re-introduced into *pdzd*-*8*^–/–^ nematodes. Nematodes were treated with 4 mM 2-DG for 2 days, followed by determination of glutaminolysis (**b**; levels of m + 5 α-ketoglutarate (α-KG) and glutamate (Glu); and m + 4 succinate (Suc), fumarate (Fum), malate (Mal), and citrate (Cit) that reflect the rates of glutaminolysis (depicted in Supplementary information, Fig. S1e) are shown), and OCR through Seahorse Analyzer (**c**, left panel). Conversely, nematode pdzd-8 or its AMPK-unphosphorylable mutant (pdzd-8-S536A) was re-introduced into *PDZD8*^–/–^ MEFs by lentiviral infection. Cells were glucose-starved for 2 h, followed by determination of OCR (**c**, right panel). See also rates of glutaminolysis in pdzd-8- and pdzd-8-S536A-re-introduced *PDZD8*^–/–^ MEFs in low glucose in Supplementary information, Fig. S1f. **d**–**g**, **i**, **j** The AMPK-PDZD8-GLS1 axis extends the lifespan of nematodes in low glucose. The *pdzd*-*8*^*–/–*^ nematodes with re-introduced PDZD8-T527A (**d**) or PDZD8-T527E (**g**, right panel; see also PDZD8-T527D re-introduction as a control in the left panel of **g**), the *aak*-*2*^–/–^nematodes with the expression of PDZD8-T527D/E (**f**), *pdzd*-*8*^–/–^ nematodes with the expression of constitutively active aak-2 (CA-*aak2*) and re-introduction of PDZD8-T527A (**e**), WT (N2) nematodes with the depletion of glna (**i**), or glna-depleted nematodes with re-introduced GLS1 (the KGA isozyme)-33A (**j**) were treated with 2-DG (**d**, **g**, **i**, **j**) or without 2-DG (**e**, **f**). Lifespan data are shown as Kaplan–Meier curves. See also the statistical analyses in Supplementary information, Table S1, and the same hereafter for all lifespan data. **h**, **k**, **l** The AMPK-PDZD8-GLS1 axis mediates CR-induced lifespan extension in nematodes. Experiments (**h**, **k**, **l**) were performed as those in **d**, **i**, **j**, except that the nematodes were subjected to CR. **m**, **n** The AMPK-PDZD8-GLS1 axis promotes pharyngeal pumping rates in nematodes. The *pdzd*-*8*^–/–^ nematodes with re-introduced PDZD8-T527A (**m**), or *glna*-null nematodes with re-introduced KGA-33A (**n**), were subjected to CR for 2 days. **o**, **p** The AMPK-PDZD8-GLS1 axis promotes resistance to oxidative stress. Nematodes were subjected to CR for 2 days, followed by treating with 15 mM FeSO_4_ (see experimental timeline on the upper (**o**); AL fed, ad libitum fed). Quenching of ROS burst period prevents the extension of lifespan (**q**). The N2 nematodes were subjected to CR. During the period of 6–120 h of CR, ROS is triggered (see Supplementary information, Fig. S3e, g), and nematodes were treated with 5 mM NAC during this period. **r**–**u** The AMPK-PDZD8-GLS1 axis plays a rejuvenating role in mice. Aged (8-month-old) *PDZD8*-MKO mice with muscle-specific re-introduction of WT PDZD8 or PDZD8-T527A were CR for 3 months, followed by determination of muscle fiber type. Representative images from whole-muscle cross sections for soleus, extensor digitorum longus (EDL), tibialis anterior (TA) and gastrocnemius (**r**). Muscular NAD^+^ levels are show in **s**. Running distance was shown in **t** (upper panel) and duration in the lower panel. Grip strength was show in **u**. Data in this figure are shown as mean ± SD (**m**, **n**, **r**–**u**) or mean ± SEM (others); *n* = 4 (**b**), or labeled on each panel; *P* values were determined by two-way ANOVA, followed by Tukey, except **c** by unpaired two-tailed Student’s *t*-test. Experiments in this figure were performed three times.

We next determined the effects of AMPK-dependent PDZD8 phosphorylation on the lifespan of *C*. *elegans*. It was previously shown that 2-DG could extend lifespan in *C*. *elegans* in an AMPK-dependent manner.^8^ Consistently, we found that knockout of *pdzd*-*8* blocked the lifespan extension induced by 2-DG, and the re-introduction of PDZD8-T527A was unable to rescue the 2-DG-induced lifespan extension in these nematodes (Fig. 1d; Supplementary information, Fig. S2a; see also statistical analyses in Supplementary information, Table S1, and the same hereafter for all lifespan data). Similarly, the lifespan-extending effects of constitutively active aak-2 (AMPKα2 homolog in *C*. *elegans*)^9^ were abrogated in the nematodes expressing PDZD8-T527A (Fig. 1e), while expression of the phospho-mimetic PDZD8-T527E extended lifespan to a similar extent to that by 2-DG, even in the nematodes lacking *aak*-*2* (Fig. 1f, g; note that the PDZD8-T527D mutant behaved rather similarly to the PDZD8-T527A, as described previously^7^), consistent with PDZD8 acting downstream of AMPK. We also cultured nematodes on agar containing diluted bacteria to mimic the AMPK-activating effect by CR^10^ (Supplementary information, Fig. S2b) and found that the increase of glutaminolysis and extension of lifespan depended on the WT PDZD8, but not PDZD8-T527A (Fig. 1h; Supplementary information, Fig. S2c, d). In addition, we found that the enhanced glutaminolysis is required for AMPK-PDZD8-mediated lifespan extension, as depletion of all the three *glna* genes (*glna*-*1* to *glna*-*3*; glutaminase homologs in *C*. *elegans*) by knockdown of *glna*-*2* in the *glna*-*1* and *glna*-*3* double knockout strain, or re-introduction of GLS1-33A that is defective in interacting with PDZD8^7^ into this *glna*-null strain, blocked lifespan extension under 2-DG or CR treatment (Fig. 1i–l). Consistently, we found that the 2-DG- and CR-extended healthspan, as evidenced by the enhancement of pharyngeal pumping rates and the resistance to severe oxidative stress induced by high concentrations of FeSO_4_, was blocked by the expression of PDZD8-T527A or GLS1-33A (Fig. 1m–p; Supplementary information, Fig. S2e–h). These data indicate that the CR-promoted extension of lifespan and healthspan in nematodes depends on the AMPK-PDZD8-GLS1 axis.

We further explored how CR-enhanced glutaminolysis promotes lifespan and healthspan. We performed a total RNA sequencing experiment and found that the expression of reactive oxygen species (ROS)-depleting enzymes, such as superoxide dismutases (SODs), was significantly increased in a p-T527-dependent manner in nematodes subjected to the treatment of 2-DG or CR (Supplementary information, Fig. S3a, b, and Table S2; see also RT-PCR data, as validation, in Supplementary information, Fig. S3c), providing a mechanistic explanation for the increased resistance to severe oxidative stress (Fig. 1o, p; Supplementary information, Fig. S2g, h). Such a linkage between p-T527-dependent increase of OCR and induction of antioxidative gene expression to lifespan extension is reminiscent of the AMPK-mediated mitohormesis, which is defined as an increase in fitness after mild mitochondrial oxidative stress (increased mitochondrial ROS) under conditions such as CR.^8,11^ Unlike severe ROS that causes constant detrimental effects, mild ROS induced by mitochondrial stress is transient and quickly eliminated by the antioxidative genes triggered by mitohormesis.^11^ Consistently, we observed a p-T527- and GLS1-dependent increase of mitochondrial ROS burst in nematodes under 2-DG or CR treatment, as assessed by the fluorescent signal of mitoSOX dye, which is intensified specifically in response to the increase in mitochondrial ROS (Supplementary information, Fig. S3d–g). We also observed an increased expression of SOD, accompanied by a leveling off of ROS, which was dependent on p-T527 (Supplementary information, Fig. S3c, f). Furthermore, administration of N-acetylcysteine (NAC; a ROS scavenger) during the period of ROS burst in calorie-restricted nematodes prevented the induction of SOD, as well as the extension of lifespan (Fig. 1q; Supplementary information, Fig. S3h).

We also examined the rejuvenating roles of the AMPK-PDZD8-GLS1 axis in mice. We found that CR sufficiently induced the phosphorylation of T527 in PDZD8 in mouse muscles without elevating AMP levels (Supplementary information, Fig. S4a, b) and led to a transient increase of mitochondrial ROS in the muscle in 8-month-old, muscle-specific *PDZD8* knockout (*PDZD8*-MKO) mice with muscle-specific re-introduction of WT PDZD8 (Supplementary Information, Fig. S4c). After three months of CR, these mice showed better muscle functions, as evidenced by an increased content of oxidative muscle fibers (Fig. 1r; determined by the expression levels of MHCI and MHCIIa, markers for oxidative muscle fibers) and decreased content of glycolytic fibers (Fig. 1r; determined by the expression levels of MHCIIb). We also observed a significant increase in muscular NAD^+^ levels in these mice (Fig. 1s). Running distance, duration, and grip strength were significantly increased in these mice (Fig. 1t, u). Such rescued phenotypes were not observed when the AMPK-unphosphorylable PDZD8-T527A was re-introduced (Fig. 1r–u). As a control, the PDZD8-T527A re-introduced mice showed similar levels of body fat and lean mass, pedestrial locomotion, and energy expenditure compared with mice with WT PDZD8 re-introduction (Supplementary information, Fig. S4d), indicating that the increased glutaminolysis in muscle caused by PDZD8 can slow down the aging process of muscle directly.

We have thus identified that the AMPK-PDZD8-GLS1 axis has anti-aging effects in both nematodes and mice as a consequence of induced glutaminolysis and mitohormesis. In addition to glutaminolysis, it is important to acknowledge that other amino acids may also play a role in inducing mitohormesis under different conditions. For instance, in *daf*-*2* knockout nematodes with impaired insulin and IGF-1 signaling (iIIS), AMPK can stimulate the catabolism of proline by upregulating the expression of _L_-proline dehydrogenase.^11^ It is also important to note that apart from AMPK-induced catabolism of amino acids, other downstream processes of AMPK activation can contribute to extending lifespan and healthspan, such as the target of rapamycin complex 1 (TORC1) inhibition, autophagy induction, and NAD^+^ elevation.^6^ Some of these events, like TORC1 inhibition and autophagy induction, can also promote amino acid catabolism by preserving the amino acid pool, for which enhanced degradation of labile proteins also plays a part.^12,13^

As AMPK and PDZD8 are ubiquitously expressed, it will be interesting to determine whether the enhanced glutaminolysis in tissues other than skeletal muscle, such as the heart, liver, and brain, contributes to lifespan extension. As the lifespan/healthspan extension is like a “barrel effect” where all the benefits mentioned above must be present for maximal effect to occur,^14^ our findings have established glutaminolysis as a critical step in the regulation of longevity, with CR being a key factor in triggering this process.

## Supplementary information

Supplementary information figure and text

Supplementary information, Table S1

Supplementary information, Table S2

Supplementary information, Table S3

Full scan

## Source data

Source data

## Data Availability

The raw RNA sequencing data corresponding to the expression of ROS-depleting enzymes in nematodes have been deposited in the Genome Sequence Archive^15^ in the National Genomics Data Center, China National Center for Bioinformation/Beijing Institute of Genomics, Chinese Academy of Sciences (GSA: CRA011002) that are publicly accessible at https://ngdc.cncb.ac.cn/gsa. Full immunoblots are provided as a “Full scans” file, and raw data and statistical analysis data are included in the “Source data” file. Any additional information required to reanalyze the data reported in this paper is available upon request. All materials generated in this study are available upon request.
