# Supplementary material for: AMPK-PDZD8-GLS1 axis mediates calorie restriction-induced lifespan extension
Source: Cell Res. 2024 Sep 19;34(11):806–9. doi: 10.1038/s41422-024-01021-3 (PMC11528062; doi:10.1038/s41422-024-01021-3)
Supplement: Supplementary file 1 — Supplementary information figure and text [file 41422_2024_1021_MOESM1_ESM.pdf]

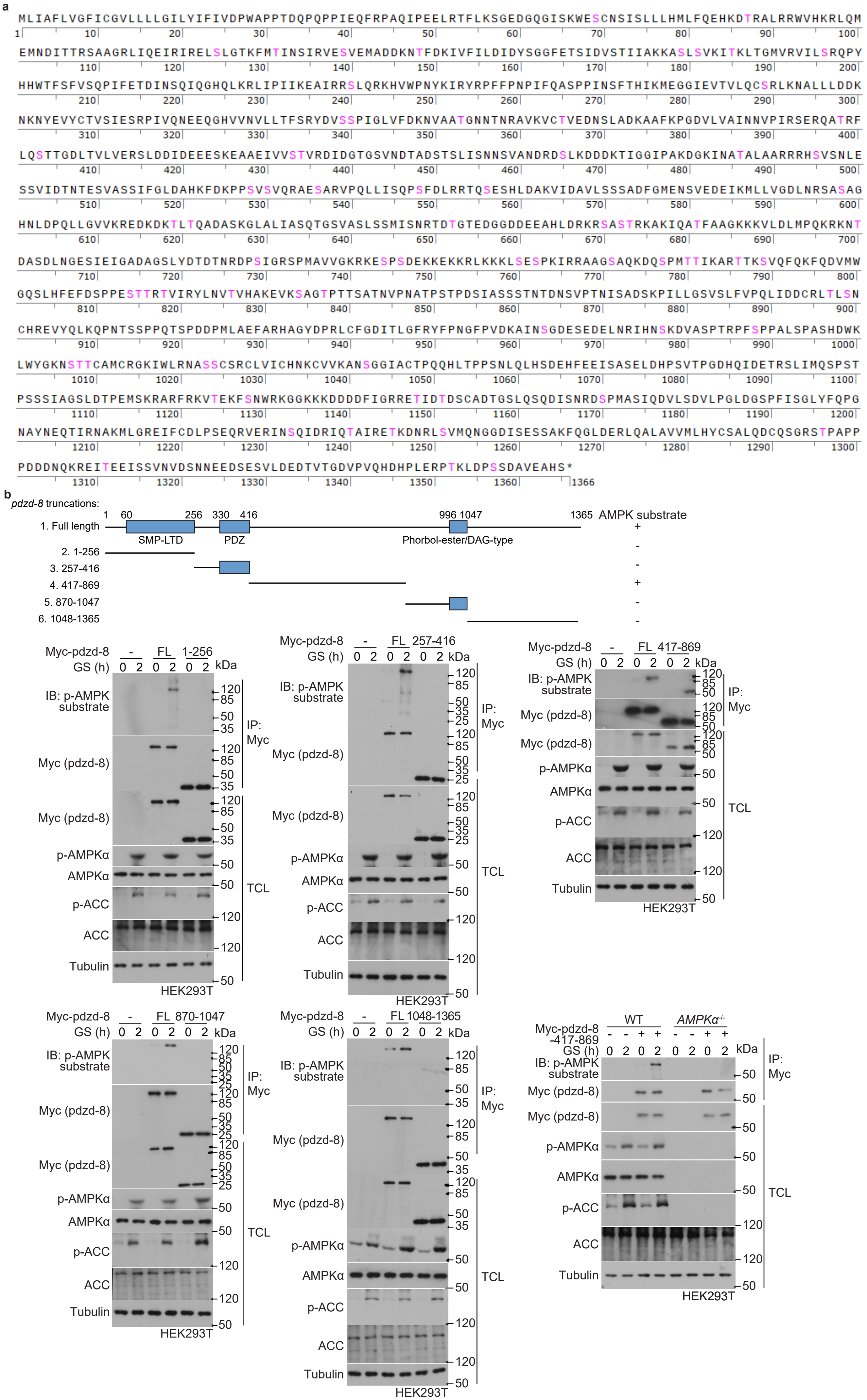

Supplementary information, Fig. S1

c

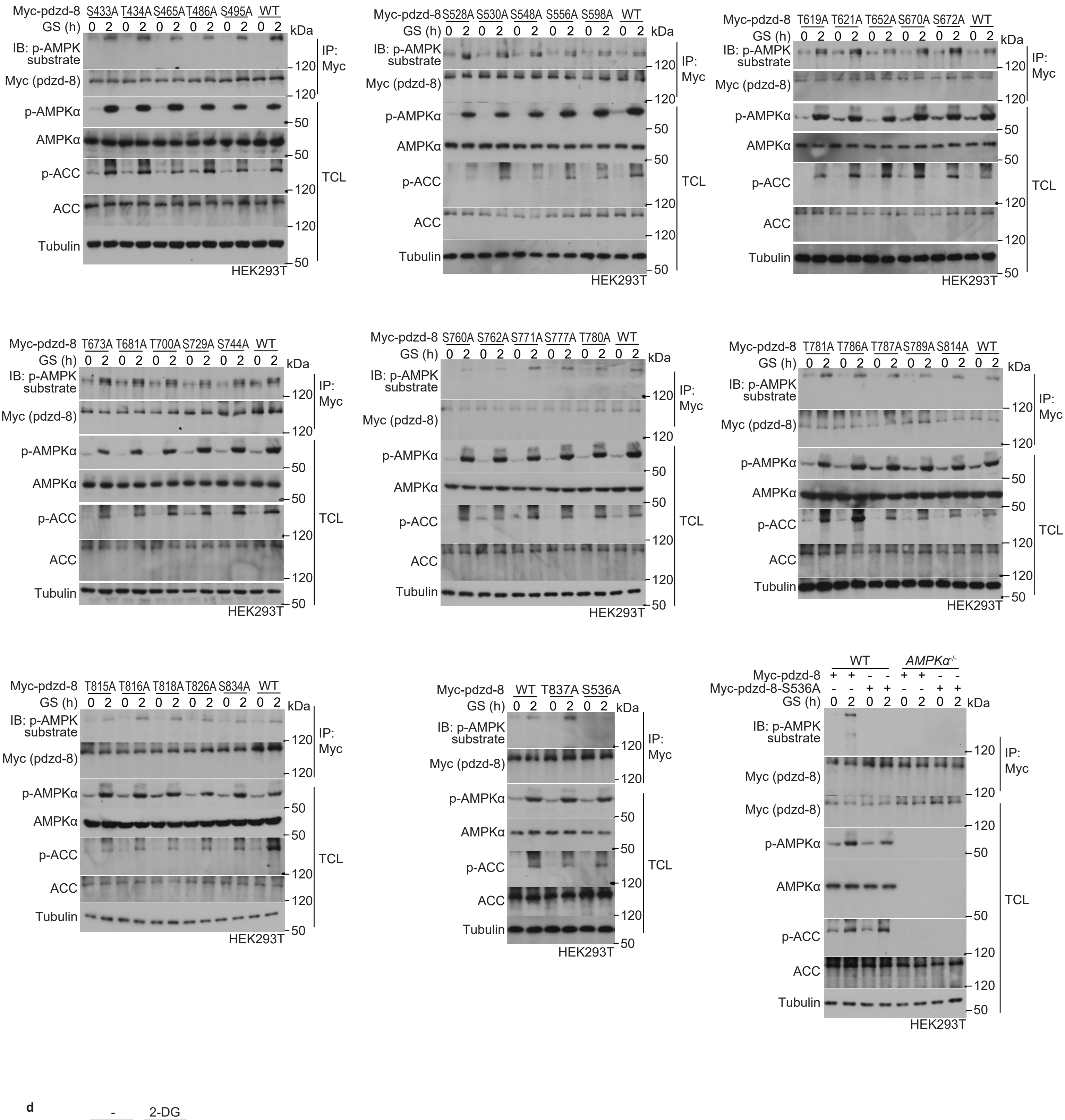

d

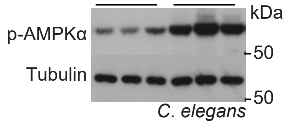

Supplementary information, Fig. S1 (Cont.)

e

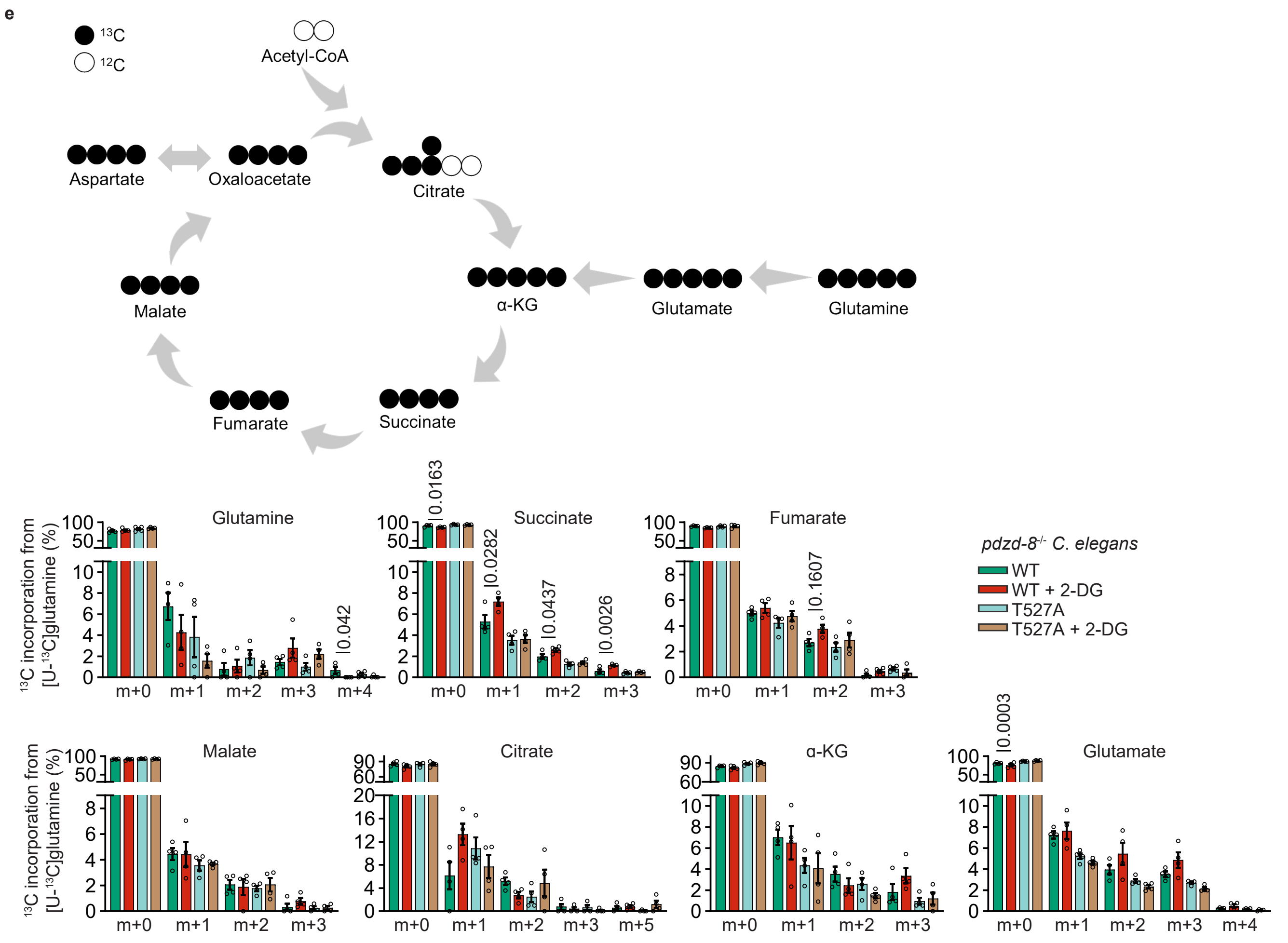

f

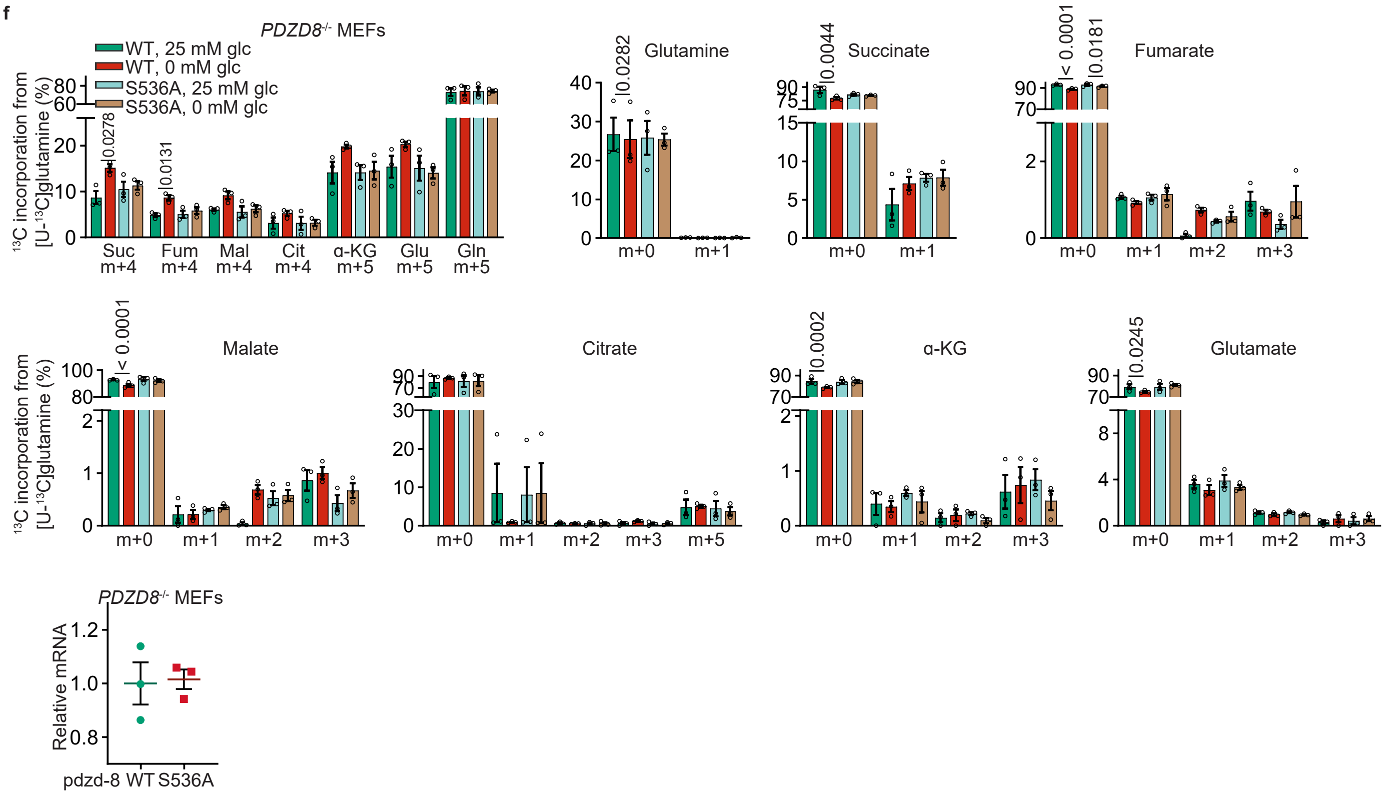

Supplementary information, Fig. S1 (Cont.)

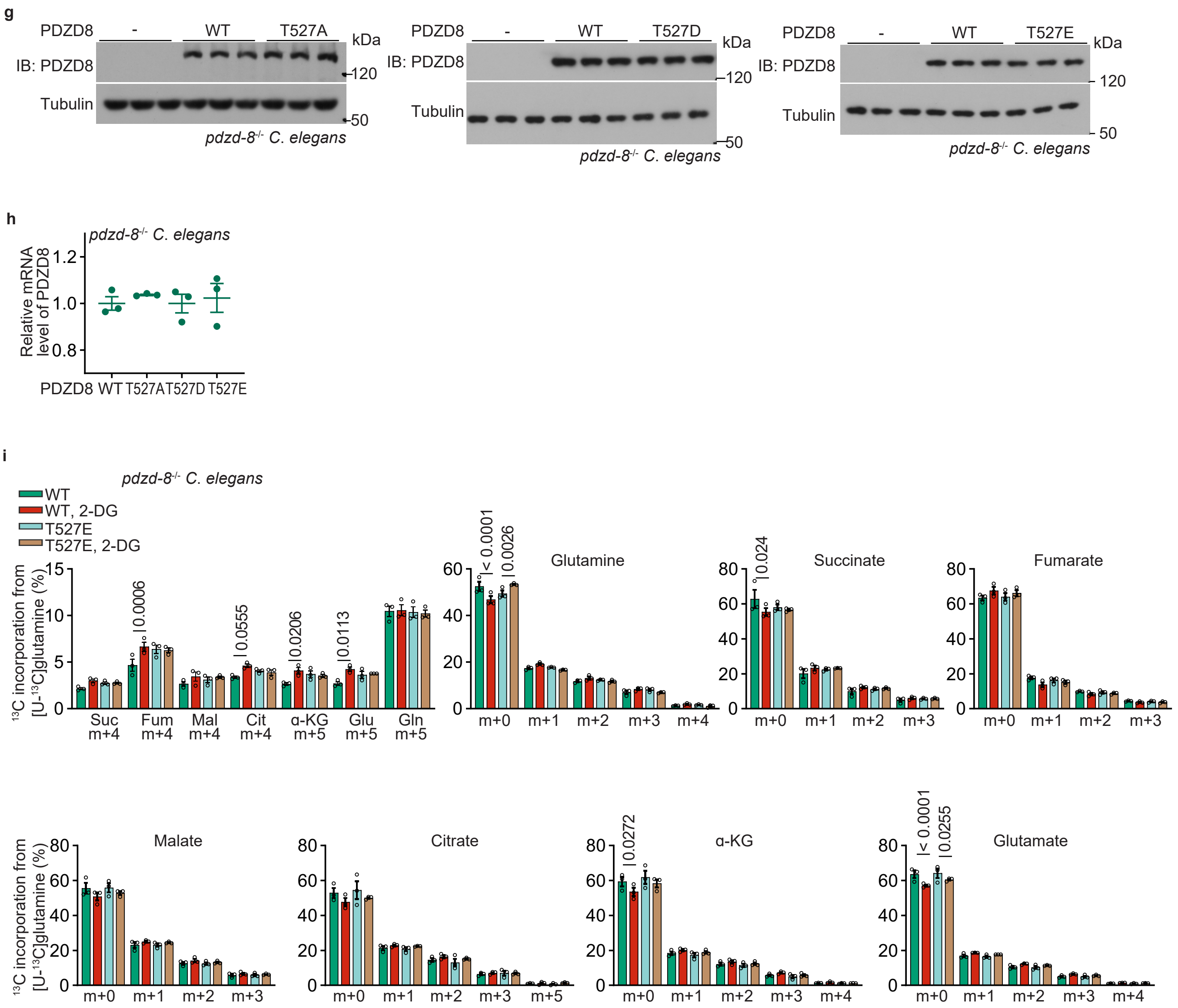

Supplementary information, Fig. S1 Nematode AMPK-PDZD8-GLS1 axis plays a conserved role in promotion of glutaminolysis in mammalian cells.

a-**c** AMPK phosphorylates the S536 site of *pdzd-8* in nematodes. HEK293T cells, either wildtype or *AMPKα*$^{-/-}$, were transfected with Myc-tagged *pdzd-8* (**b**, **c**), *pdzd-8* deletions (**b**), or *pdzd-8* point mutations (**c**), and then glucose-starved for 2 h. Cells were then lysed, followed by immunoprecipitation of Myc-tag and immunoblotting using the pan-phospho-AMPK-substrates antibody. See also the potential phosphoacceptor sites (colored in magenta) that fit the conserved motif of the AMPK substrates of *pdzd-8* in **a**. FL, full length.

d Fasting activates nematodes AMPK. Nematodes were treated with 4 mM 2-DG that mimics fasting, for 2 days, and the AMPK (aak-2) phosphorylation was determined by immunoblotting.

e Human PDZD8-T527A blocks fasting-induced glutaminolysis in nematodes. The *pdzd-8*$^{-/-}$ nematodes with re-introduced human PDZD8 or PDZD8-T527A were treated as in Fig. 1b. Data are shown as mean ± SD; n = 4 biological replicates for each condition/genotype; p values were calculated by two-way ANOVA, followed by Tukey. Levels of other isotopomers of the labeled TCA cycle intermediates are shown in Fig. 1b.

f Nematode pdzd-8-S536A blocks fasting-induced glutaminolysis in mammalian cells. The *PDZD8*$^{-/-}$ MEFs with pdzd-8 or pdzd-8-S536A stably expressed (the mRNA levels of pdzd-8 and pdzd-8-S536A were confirmed using RT-PCR and are shown in the lower panel as mean ± SEM; n = 3 samples for each gene; p values were determined by two-way ANOVA) were glucose-starved for 2 h, followed by determination of glutaminolysis through analyzing the levels of labeled TCA cycle intermediates by GC-MS. Data are shown in the upper panel as mean ± SEM; n = 4 samples for each condition. p values were determined by two-way ANOVA, followed by Tukey.

g, **h** Validation of the expression levels of PDZD8 and its mutant T527A, T527D, and T527E when re-introduced into *pdzd-8*$^{-/-}$ nematodes by immunoblotting (**g**) and RT-PCR (**h**; data shown as mean ± SEM; n = 3 samples for each gene; p values were determined by two-way ANOVA, followed by Tukey).

i PDZD8-T527E promotes glutaminolysis regardless of low glucose. Experiments were performed as in Fig. 1b, except that *pdzd-8*$^{-/-}$ nematodes with human PDZD8-T527E mutant re-introduced were used. Data are shown as mean ± SEM; n = 4 samples for each condition. p values were determined by two-way ANOVA, followed by Tukey.

Experiments in this figure were performed three times.

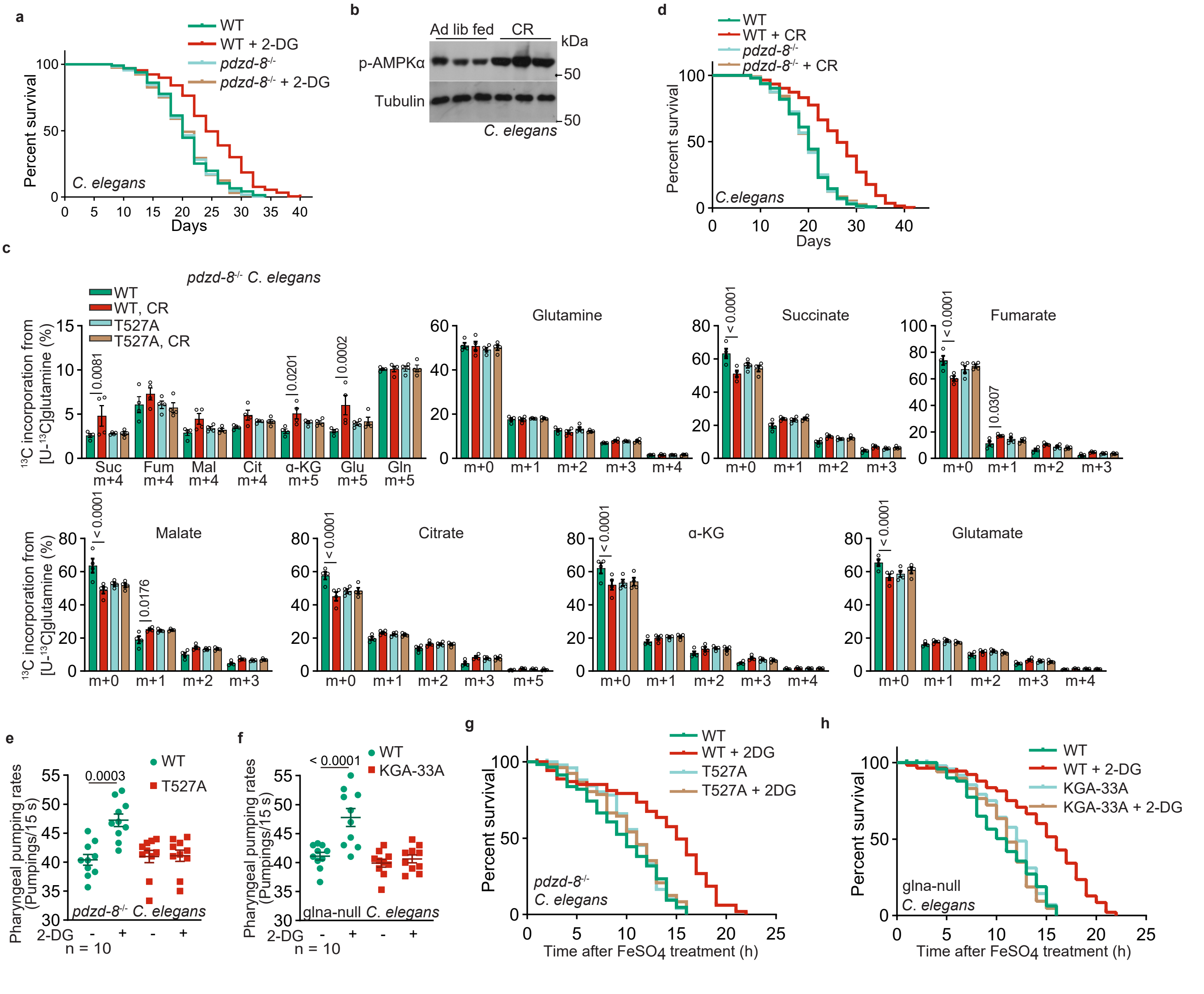

Supplementary information, Fig. S2 AMPK-PDZD8-GLS1 axis exerts rejuvenating effects in nematodes

a, **d** PDZD8 extends the lifespan of nematodes in low glucose. The *pdzd-8*$^{-/-}$ nematodes were treated with 2-DG (**a**) or subjected to CR (**d**). Lifespan data are shown as Kaplan-Meier curves.

b CR activates nematode AMPK. Nematodes were subjected to CR for 2 days, and the AMPK (aak-2) phosphorylation was determined by immunoblotting.

c PDZD8-T527A blocks CR-induced glutaminolysis in nematodes. The *pdzd-8*$^{-/-}$ *C. elegans* strains with re-introduced human PDZD8 or PDZD8-T527A were subjected to CR for 2 days, followed by determination of glutaminolysis through analyzing the levels of labeled TCA cycle intermediates by GC-MS. Data are shown as mean ± SD; n = 4 biological replicates for each condition/genotype; p values were calculated by two-way ANOVA, followed by Tukey.

e, **f** AMPK-PDZD8-GLS1 axis promotes pharyngeal pumping rates in nematodes. Experiments were performed as in Fig. 1m (**e**) and 1n (**f**), respectively, except that nematodes were treated with 2-DG for 2 days. Data are shown as mean ± SD; n = 10 for each condition/genotype. p values were calculated by two-way ANOVA, followed by Tukey.

g, **h** AMPK-PDZD8-GLS1 axis promotes resistance of nematodes to oxidative stress. Experiments were performed as in Fig. 1o (**g**) and 1p (**h**), respectively, except that nematodes were treated with 2-DG for 2 days before the $FeSO_4$ treatment.

Experiments in this figure were performed three times.

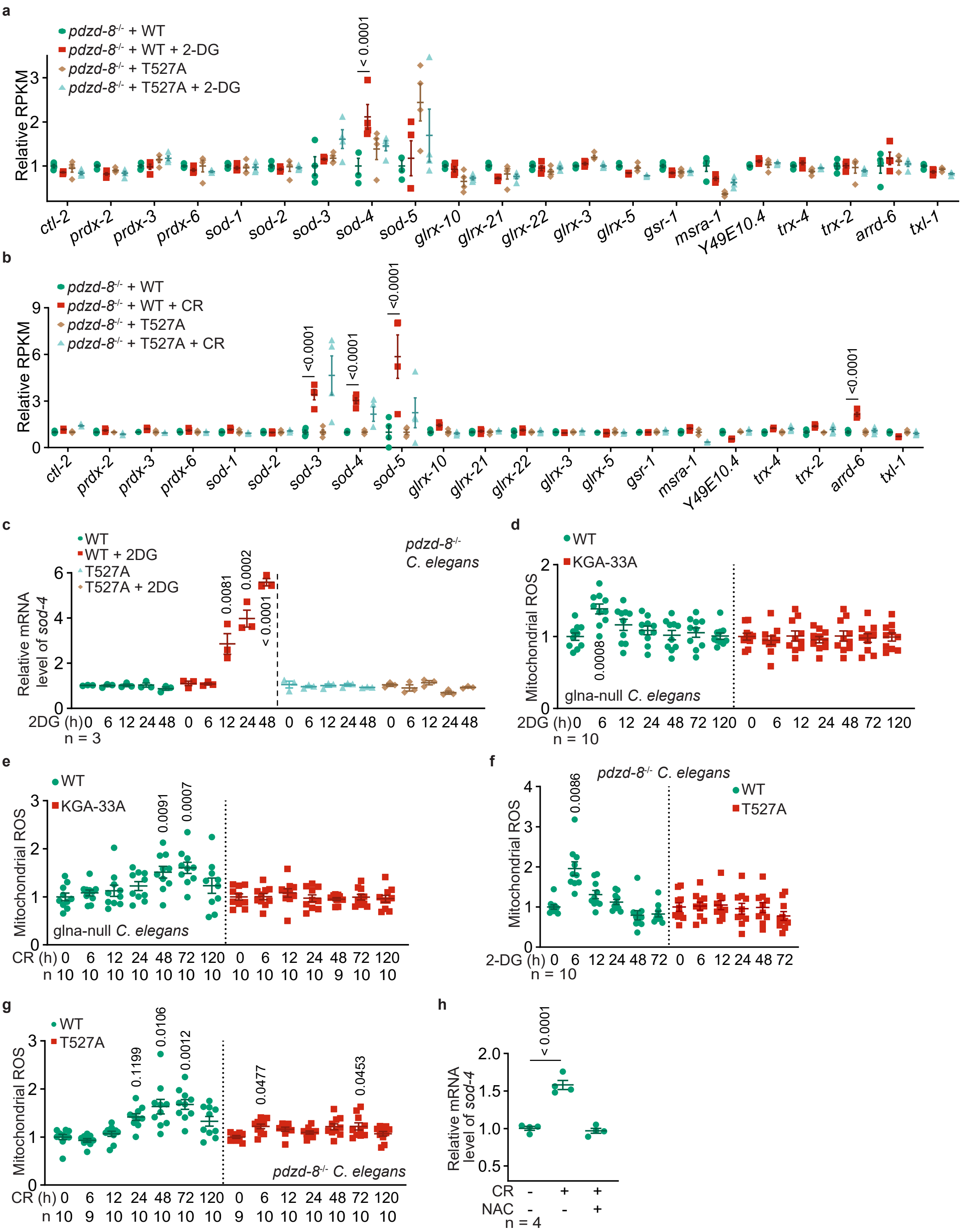

Supplementary information, Fig. S3 PDZD8 exerts rejuvenating effects in nematodes

a, f AMPK-PDZD8 axis induces transient mitochondrial ROS and expression of ROS-depleting enzymes in low glucose. The *pdzd-8*$^{-/-}$ nematodes with re-introduced PDZD8-T527A were treated with 2-DG for desired durations, followed by determination of mitochondrial ROS using the mitoSOX dye (**f**; data are shown as mean ± SEM; n = 10 biological replicates for each condition). At 48 h after 2-DG treatment, an RNA-sequencing experiment was performed, and the mRNA levels of ROS-depleting enzymes are shown (**a**; data are shown as mean ± SEM; n = 4 biological replicates for each condition/genotype). p values were determined by one-way ANOVA, followed by Dunn (WT nematodes of **f**) or Tukey (T527A nematodes of **f**), or by two-way ANOVA, followed by Tukey (**a**), all compared to the untreated group of each genotype.

b, g AMPK-PDZD8 axis induces transient mitochondrial ROS and expression of ROS-depleting enzymes in nematodes subjected to CR for the desired duration (**g**, or 2 days in **b**). Experiments in (**g**) (data are shown as mean ± SEM; with n values labeling on the panel) and (**b**) (data are shown as mean ± SEM; n = 4 biological replicates for each condition/genotype) were performed as in **f** and **a**, except CR was applied. p values were determined by one-way ANOVA, followed by Dunn (WT nematodes of **g**) or Tukey (T527A nematodes of **g**), or by two-way ANOVA, followed by Tukey (**b**), all compared to the untreated group of each genotype.

c AMPK-PDZD8 axis is required for the increased expression of sod-4. The *pdzd-8*$^{-/-}$ *nematodes* with re-introduced wildtype PDZD8 or PDZD8-T527A were treated with 2-DG for desired durations, followed by determination of mRNA levels of *sod-4* by RT-PCR. Data are shown as mean ± SEM; n = 3 biological replicates for each condition/genotype. p values were calculated by one-way ANOVA, followed by Tukey, all compared to the untreated group of each genotype.

d, e GLS1 is required for the AMPK-PDZD8-induced transient mitochondrial ROS in nematodes. Experiments were performed as in **f** and **g**, respectively, except that the glna-depletion strain re-introduced with KGA-33A was used. Data are shown as mean ± SD; n values are labeled on each panel. p values were calculated by two-way ANOVA, followed by Tukey, all compared to the untreated group of each genotype.

h Quenching of ROS burst in calorie-restricted nematodes prevents the induction of ROS-depleting enzymes. The N2 nematodes were subjected to CR and were treated with 5 mM NAC, as in Fig. 1q. The mRNA levels of *sod-4* were determined by RT-PCR. Data are shown as mean ± SEM; n = 3 biological replicates for each condition. p values were calculated by one-way ANOVA, followed by Tukey.

Experiments in this figure were performed three times.

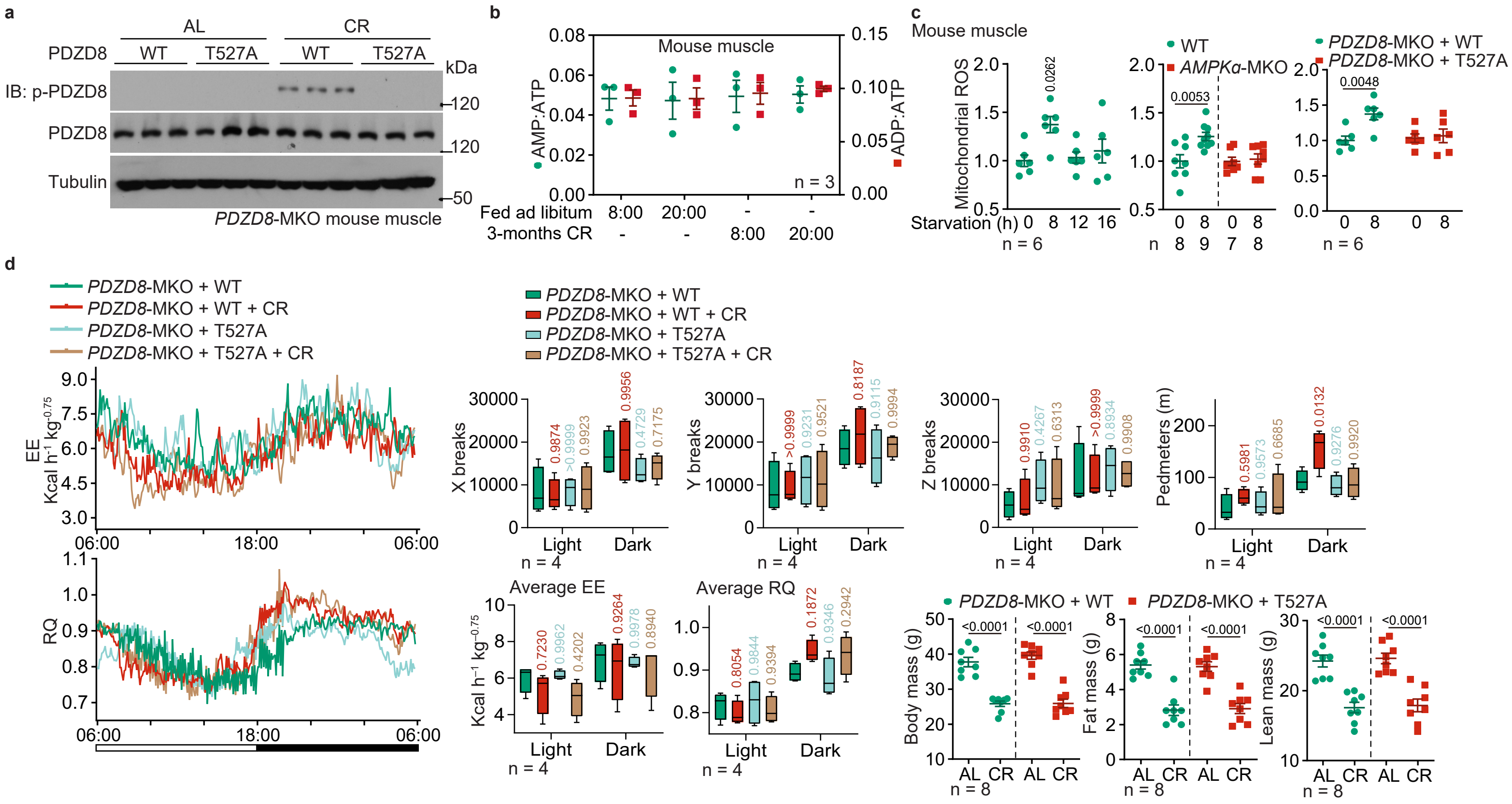

Supplementary information, Fig. S4 PDZD8 exerts rejuvenating effects in mice

a Validation of PDZD8 phosphorylation in the *PDZD8*-MKO mice with muscle-specific re-introduction of wildtype PDZD8 or PDZD8-T527A. Mice at 8 months were subjected to CR for another 3 months, followed by immunoblotting to determine PDZD8-phosphorylation in muscle tissues at 4 p.m. (1 h before the feeding time of each day during CR).

b CR does not elevate AMP levels in mouse muscle. *PDZD8*-MKO mice with muscle-specific re-introduction of wildtype PDZD8 were subjected to CR or fed ad libitum for 3 months, followed by determining the AMP:ATP and ADP:ATP ratios by CE-MS. Data are shown as mean ± SEM; n = 3 mice for each condition; and p values were determined by one-way ANOVA, followed by Tukey.

c AMPK-PDZD8 axis induces transient mitochondrial ROS in mouse muscle. Wildtype mice (left panel), *AMPKα*-MKO mice (middle panel), or *PDZD8*-MKO mice with muscle-specific re-introduction of wildtype PDZD8 or PDZD8-T527A (right panel) were subjected to CR, followed by determination of muscle mitochondrial ROS using the mitoSOX dye at different starvation time points during the first day of CR. Data are shown as mean ± SEM; n (labeled on each panel) values indicate biological replicates for each condition; and p values were determined by one-way ANOVA, followed by Tukey (left panel), unpaired two-tailed Student's *t*-test (middle panel), or two-way ANOVA, followed by Sidak (right panel).

d Muscle-specific re-introduction of PDZD8 does not affect body, fat and lean mass, pedestrial locomotion, or energy expenditure. Aged (8-month-old) *PDZD8*-MKO mice with muscle-specific re-introduction of wildtype PDZD8 or PDZD8-T527A were subjected to CR for 3 months, followed by determination of body weight, body composition (fat and lean mass), and energy expenditure (EE; see also respiratory quotient (RQ) and locomotion data). Data are shown as the mean ± SEM (body weight and body composition), mean (EE curves; at 5-min intervals during a 24-h course after normalization to the body weight ($kg^{0.75}$), and RQ curves), or as box-and-whisker plot (average EE, average RQ, and locomotion, in which the lower and upper bounds of the box represent the first and the third quartile scores, the center line represents the median, and the lower and upper limits denote minimum and maximum scores, respectively); n = 4 (EE, RQ, and locomotion) or 8 (others) mice for each treatment; and p values were determined by one-way ANOVA, followed by Tukey (body, fat and lean mass), two-way ANOVA, followed by Sidak (average EE), or two-way ANOVA, followed by Dunnet (others).

Experiments in this figure were performed three times.

MATERIALS AND METHODS

Antibodies

Rabbit polyclonal antibody against p-T527-PDZD8 (1:1,1000 dilution for immunoblotting (IB)) was raised and validated as described previously[1]. Rabbit anti-phospho-AMPKα-Thr172 (cat. #2535, RRID: AB_331250; 1:1,000 for IB), anti-AMPKα (cat. #2532, RRID: AB_330331; 1:1,000 for IB), anti-phospho-AMPK substrate motif (cat. #5759, RRID: AB_10949320; 1:1,000 for IB and 1:25 for immunoprecipitation (IP)) anti-phospho-ACC-Ser79 (cat. #3661, RRID: AB_330337; 1:1,000 for IB), anti-ACC (cat. #3662, RRID: AB_2219400; 1:1,000 for IB), anti-Myc-tag (cat. #2278, RRID: AB_490778; 1:1,000 for IB and 1:100 for IP), horseradish peroxidase (HRP)-conjugated mouse anti-rabbit IgG (conformation-specific, cat. #5127, RRID: AB_10892860; 1:2,000 for IB), and mouse anti-Myc-tag (cat. #2276, RRID: AB_331783; 1:500 for IB and 1:100 for IP) antibodies were purchased from Cell Signaling Technology. Rabbit anti-PDZD8 (cat. NBP2-58671; 1:1,000 for IB) was purchased from Novus Biologicals. Rabbit anti-tubulin (cat. 10068-1-AP, RRID: AB_2303998; 1:1,000 for IB nematode tubulin) and mouse anti-tubulin (cat. 66031-1-Ig, RRID: AB_11042766; 1:20,000 for IB mammalian tubulin) antibodies were purchased from Proteintech. Mouse anti-MHCIIa (cat. SC71, RRID: AB_2147165; 1:100 for immunohistochemistry (IHC)), anti-MHCIIb (cat. BF-F3, RRID: AB_2266724; 1:100 for IHC), and anti-MHCI (cat. C6B12, RRID: AB_528351; 1:100 for IHC) antibodies were purchased from Developmental Studies Hybridoma Bank. HRP-conjugated goat anti-mouse IgG (cat. #115-035-003, RRID: AB_10015289;

1:5,000 for IB) and goat anti-rabbit IgG (cat. #111-035-003, RRID: AB_2313567; 1:5,000 for IB) antibodies were purchased from Jackson ImmunoResearch.

Chemicals and assay kits

Glucose (cat. G7021), DMSO (cat. D2650), PBS (cat. P5493), NaCl (cat. S7653), NaOH (cat. S8045), HCl (cat. 320331), agar (cat. A1296), $CaCl_2$ (cat. C5670), $MgSO_4$ (cat. M2643), KH_2PO_4 (cat. P5655), K_2HPO_4 (cat. P9666), cholesterol (cat. C3045), Na_2HPO_4 (cat. S7907), NaH_2PO_4 (cat. S8282), sodium hypochlorite solution (NaClO; cat. 239305), HEPES (cat. H4034), EDTA (cat. E6758), EGTA (cat. E3889), $MgCl_2$ (cat. M8266), CsCl (cat. 289329), ethanol (cat. 459836), isopropanol (cat. 34863), glycerol (cat. G5516), IGEPAL CA-630 (NP-40, cat. I3021), Triton X-100 (cat. T9284), Tween-20 (cat. P9416), dithiothreitol (DTT; cat. 43815), IPTG (cat. I6758), carbenicillin (cat. C1613), streptomycin (for nematode culture; cat. 85886), Trioxsalen (TMP; cat. T6137), 2-deoxy-D-glucose (2-DG; cat. D8375), ampicillin (cat. A9518), kanamycin (cat. E004000), iron(II) sulfate heptahydrate ($FeSO_4$; cat. F8633), agarose (cat. A9539), hexadimethrine bromide (polybrene; cat. H9268), triple-free DMEM (cat. D5030), sodium palmitate (PA; cat. P9767), carnitine (cat. C0283), Trizma base (Tris; cat. T1503), sodium pyrophosphate (cat. P8135), β-glycerophosphate (cat. 50020), sodium azide (NaN_3; cat. S2002), oligomycin A (cat. 75351), FCCP (cat. C2920), antimycin A (cat. A8674), rotenone (cat. R8875), myristic-d27 acid (cat. 68698), BSA (cat. A2153), methoxyamine hydrochloride (cat. 89803), MTBSTFA (with 1% t-BDMCS; cat. M-108), pyridine (cat. 270970), methanol (cat. 646377), chloroform (cat.

C7559), ammonium acetate (cat. 73594), ammonium hydroxide solution (cat. 338818), LC-MS-grade water (cat. 1153332500), mannitol (cat. M4125), L-methionine sulfone (cat. M0876), D-campher-10-sulfonic acid (cat. 1087520), 3-aminopyrrolidine dihydrochloride (cat. 404624), N,N-diethyl-2-phenylacetamide (cat. 384011), trimesic acid (cat. 482749), diammonium hydrogen phosphate (cat. 1012070500), ammonium trifluoroacetate (cat. 56865), FCCP (cat. C2920), and NAC (N-acetylcysteine; cat. A9165) were purchased from Sigma. Penicillin-streptomycin (for DMEM preparation; cat. 15140163), TRIzol (cat. 15596018), UltraPure DNase/RNase-Free Distilled Water (RNase-free water; cat. 10977015), Maxima SYBR Green/ROX qPCR master mix (cat. K0223), RNase-free DNase I (cat. EN0523), Qubit RNA BR assay kit (cat. Q10211), Collibri Stranded RNA Library Prep Kit (cat. A39003024), Collibri Library Quantification Kit (cat. A38524100), DMEM, high glucose (DMEM; cat. 11965175), glucose-free DMEM (cat. 11966025), FBS (cat. 10099141C), Lipofectamine 2000 (cat. 11668500), MEM non-essential amino acids solution (cat. 11140050), GlutaMAX (cat. 35050061), sodium pyruvate (cat. 11360070), MitoSOX (cat. M36008), and Prestained Protein MW Marker (cat. 26612) were purchased from Thermo Scientific. Bacteriological peptone (cat. LP0137) was purchased from Oxoid. Seahorse XF base medium (cat. 103334) and Seahorse XF Calibrant solution (cat. 100840) were purchased from Agilent. O.C.T. Compound (cat. 4583) was purchased from Sakura Finetek USA, Inc. PrimeSTAR HS polymerase (cat. R40A) was purchased from Takara. Polyethylenimine (PEI; cat. 23966) was purchased from Polysciences. Nonfat dry milk (cat. #9999) and normal goat serum (NGS; cat. #5425) were purchased from Cell

Signaling Technology. ReverTra Ace qPCR RT master mix with a gDNA Remover kit was purchased from TOYOBO. Protease inhibitor cocktail (cat. 70221) and KAPA HyperPure magnetic beads (cat. KK8010) were purchased from Roche. WesternBright ECL and peroxide solutions (cat. 210414-73) were purchased from Advansta. [U-^{13}C]-glutamine (cat. 184161-19-1) was purchased from Cambridge Isotope Laboratories. The isotope-labeled AMP (cat. 123603801), ADP (cat. 129603601), and ATP (cat. 121603801) standards were purchased from Silantes. 3-hydroxynaphthalene-2,7-disulfonic acid disodium salt (2-naphtol-3,6-disulfonic acid disodium salt; cat. H949580) was purchased from Toronto Research Chemicals. Hexakis(1H,1H,3H-perfluoropropoxy)phosphazene (hexakis(1H, 1H, 3H-tetrafluoropropoxy)phosphazine; cat. sc-263379) was purchased from Santa Cruz Biotechnology. rProtein A Sepharose Fast Flow (cat. 17127904) and Protein G Sepharose 4 Fast Flow (cat. 17061806) beads were purchased from Cytiva.

Mouse strains

Protocols for all rodent experiments were approved by the Institutional Animal Care and the Use Committee of Xiamen University (XMULAC20180028 and XMULAC20220050). Wildtype C57BL/6J mice (#000664) were obtained from The Jackson Laboratory. *PDZD8*-MKO mice with skeletal muscle-specific reintroduction of PDZD8 or its T527A mutant were generated as described previously[1].

The following ages of mice were used: 1) for analyzing ROS in mouse skeletal muscles:

wild-type, *AMPKα*-MKO, and *PDZD8*-MKO mice with or without wildtype PDZD8 or PDZD8-T527A re-introduced, aged 8 weeks; and 2) for determining rejuvenating effects of CR: wild-type, and *PDZD8*-MKO mice with or without wildtype PDZD8 or PDZD8-T527A re-introduced, aged 32 weeks.

Caenorhabditis elegans **strains**

Nematodes (hermaphrodites) were maintained on NGM plates spread with *E. coli* OP50 as standard food[2]. All worms were cultured at 20 °C. Wildtype (N2 Bristol), *aak-2*(ok524; *aak-2*$^{-/-}$), and CA-*aak-2* (AGD467; ref. [3]) strains were obtained from *Caenorhabditis* Genetics Center, and *glna-1* (tm6647) and *glna-3* (tm8446) from National BioResource Project (NBRP). See also the details of all the nematode strains used in this study in Supplementary Information, Table S3. All mutant strains were outcrossed 6 times to N2 before the experiments. Unless stated otherwise, worms were maintained on nematode growth medium (NGM) plates (1.7% (w/v) agar, 0.3% (w/v) NaCl, 0.25% (w/v) bacteriological peptone, 1 mM $CaCl_2$, 1 mM $MgSO_4$, 25 mM KH_2PO_4-K_2HPO_4, pH 6.0, 0.02% (w/v) streptomycin and 5 μg/mL cholesterol) spread with *Escherichia coli* OP50 as standard food.

The *glna-1*-knockout and *glna-3*-knockout strains were crossed to generate a *glna-1* and *glna-3* double knockout strain (as an example, and similar procedures were applied to generate the CA-*aak-2*;*pdzd-8*$^{-/-}$ strain). Before crossing, *glna-1*-knockout hermaphrodites were synchronized: worms were washed off from agar plates with 15

mL M9 buffer (22.1 mM KH_2PO_4, 46.9 mM Na_2HPO_4, 85.5 mM NaCl and 1 mM $MgSO_4$; final concentration, and same hereafter) supplemented with 0.05% (v/v) Triton X-100 per plate, followed by centrifugation at 1,000× *g* for 2 min. The worm sediments were suspended with 6 mL of M9 buffer containing 50% synchronizing bleaching solution (by mixing 25 mL of NaClO solution (5% active chlorine), 8.3 mL of 25% (w/v) NaOH, and 66.7 mL of M9 buffer, for a total of 100 mL), followed by vigorous shaking for 2 min and centrifugation for 2 min at 1,000× *g*. The sediments were washed twice with 12 mL of M9 buffer, then suspended with 6 mL of M9 buffer, followed by rotating at 20 °C, 30 r.p.m. for 12 h. The synchronized worms were then transferred to the NGM plate and cultured to the L4 stage, followed by heat-shocking at 28 °C for 12 h. The heat-shocked worms were then cultured at 20 °C for 4 days, and the males were picked up for mating with *glna-1*-knockout hermaphrodites for another 36 h. The mated hermaphrodites were transferred to new NGM plates and allowed to give birth to more *glna-1*-knockout males for another 4 days at 20 °C. The *glna-1*-knockout males were then picked up and co-cultured with *glna-3*-knockout hermaphrodites at a 1:2 ratio (e.g., 4 males and 8 hermaphrodites on a 10-cm NGM plate) for mating for 36 h at 20 °C, and the mated hermaphrodites (*glna-3*-knockout) were picked up for culturing for another 2 days. The offspring were then picked up and individually cultured on the 35-mm NGM plate, then individually subjected to genotyping after egg-laying (after culturing for approximately 2 days). For genotyping, individual worms were lysed with 5 μl of Single Worm lysis buffer (50 mM HEPES, pH 7.4, 1 mM EGTA, 1 mM $MgCl_2$, 100 mM KCl, 10% (v/v) glycerol, 0.05% (v/v) NP-40, 0.5 mM DTT and protease

inhibitor cocktail). The lysates were then frozen at -80 °C for 12 h, then incubated at 65 °C for 1 h and 95 °C for 15 min on a thermocycler (XP Cycler, Bioer). The lysates were then cooled to room temperature, followed by amplifying genomic DNA on a thermocycler with the following programs: pre-denaturing at 95 °C for 10 min; denaturing at 95 °C for 10 s, then annealing and extending at 60 °C for 30 s in each cycle; cycle number: 35. The following primer pairs were used for identifying the *glna-1*-knockout: 5'-CCTGGACTGGGAATCGTTCA-3' and 5'-TACAACTGCGAAACACCGAG-3'; and 5'-CCCTCATTATGCGAACGAAC-3' and 5'-CCCCCAGAAGTAGATAAACG-3' for identifying the *glna-3*-knockout. The offspring generated from *glna-1*- and *glna-3*-knockout-assured individuals were then outcrossed six times to the N2 strain.

The *glna-2* was then knocked down in the *glna-1* and *glna-3* double knockout strain following the previously described procedures[4]. Briefly, synchronized worms (around the L1 stage) were placed on the RNAi plates (NGM containing 1 mg/mL IPTG and 50 μg/mL carbenicillin) spread with HT115 *E. coli* stains containing RNAi against *glna-2* (well L20 on plate II-5 from the Ahringer *C. elegans* RNAi Collection) for 2 days. The knockdown efficiency was then examined by determining the levels of *glna-2* mRNA by real-time quantitative PCR (qPCR). Approximately 1,000 worms were washed off from an RNAi plate with 15 mL of M9 buffer containing Triton X-100, followed by centrifugation for 2 min at 1,000× *g*. The sediment was washed twice with 1 mL of M9 buffer and then lysed with 1 mL of TRIzol. The worms were then frozen

in liquid nitrogen, thawed at room temperature, and then subjected to repeated freeze-thaw for another two times. The worm lysates were then placed at room temperature for 5 min, mixed with 0.2 mL of chloroform, followed by vigorous shaking for 15 s. After 3 min, lysates were centrifuged at 20,000× *g* at 4 °C for 15 min, and 450 µl of the aqueous phase (upper layer) was transferred to a new RNase-free centrifuge tube (Biopur, Eppendorf), followed by mixing with 450 µl of isopropanol, then centrifuged at 20,000× *g* at 4 °C for 10 min. The sediments were washed with 1 mL of 75% ethanol (v/v) followed by centrifugation at 20,000× *g* for 10 min, then with 1 mL of anhydrous ethanol followed by centrifugation at 20,000× *g* for 10 min. The sediments were then dissolved with 20 µl of RNase-free water after evaporating the ethanol. The dissolved RNA was then reverse-transcribed to cDNA using ReverTra Ace qPCR RT master mix with a gDNA Remover kit, followed by performing real-time qPCR using Maxima SYBR Green/ROX qPCR master mix on a CFX96 thermocycler (Bio-Rad) with the programs as described in genotyping the *glna*-knockout strain. Data were analyzed using CFX Manager software (v.3.1, Bio-Rad). Knockdown efficiency was evaluated according to the CT value obtained. The primers for *glna-2* are 5’-ACTGTTGATGGTCAAAGGGCA-3’ and 5’-CTTGGCTCCTGCCCAACATA-3’. The primers for *ama-1* (the internal control) are 5’-GACATTTGGCACTGCTTTGT-3’ and 5’-ACGATTGATTCCATGTCTCG-3’.

The *pdzd-8*$^{-/-}$ nematode strains expressing human PDZD8 or its T527A mutant were established following the methods described previously[4], with minor modifications: a)

PDZD8-WT or its T527A mutant was first introduced to the N2 strain; b) such generated strains were then subjected to knock out of the *pdzd8* gene; and c) the *pdzd8*-knockout worms were then picked up for the further outcrossing with N2 strain. Briefly, to generate an N2 strain expressing PDZD8 or its T527A mutant, cDNA of PDZD8 or PDZD8-T527A mutant was inserted into a pJM1 vector, with GFP as a selection marker between the *Nhe* I and *Kpn* I sites (expressed under control by a *sur-5* promoter), then injected into the syncytial gonad of the worm (200 ng/μL, 0.5 μL per worm). The injected worms were then recovered on an NGM plate for 2 days, and the F_1 GFP-expressing hermaphrodites were selected for further culture. The extrachromosomally existing PDZD8 or PDZD8-T527A expression plasmid was then integrated into the nematode genome using UV irradiation to establish nonmosaic transgenic strains described previously[5], with minor modifications. Briefly, 70 PDZD8 or PDZD8-T527A mutant-expressing worms at the L4 stage were picked up and incubated with 600 μL of M9 buffer, followed by adding 10 μL of TMP solution (3 mg/mL stock concentration in DMSO) and rotating at 30 r.p.m. for 15 min in the dark. Worms were then transferred to a 10-cm NGM plate without OP50 bacteria in the dark, followed by irradiating with UV at a total dose of 35 J/cm^2 (exposed within 35 s) on a UV crosslinker (CL-508; UVITEC). The irradiated worms were fed with 1 mL of OP50 bacteria at 10^{13}/mL concentration, and then cultured at 20 °C for 5 h in the dark, followed by individually cultured on a 35-mm NGM plate for 1 week without transferring to any new NGM plate (to make sure that F_1 was under starvation before further selection). The F_1 GFP-expressing hermaphrodites were selected and individually cultured for another 2 days,

and those F_2 with 100% GFP-expressing hermaphrodites were selected for further culture. The genomic sequence encoding *pdzd-8* was then knocked out from this strain by injecting a mixture of a pDD122 (Peft-3::Cas9 + ttTi5605 sgRNA) vector carrying sgRNAs against *pdzd-8* (5'-GAGGATCGTATCCAGCATGG-3', and 5'-GTGAGCACGAAGAAGCGTTG-3', designed using the CHOPCHOP website http://chopchop.cbu.uib.no/), into young adult worms. The F_1 hermaphrodite worms were individually cultured on an NGM plate. After egg-laying, worms were lysed using Single Worm lysis buffer, followed by PCR with the programs as described in genotyping the *glna*-knockout strain, except that the primers 5'-ATCTCCACCACAAACATCACCT-3' and 5'-CTTCAAAATGCTCGTCAGAGTG-3' were used. The offspring generated from knockout-assured individuals were outcrossed six times to the N2 strain. The *pdzd-8*$^{-/-}$ nematode strains expressing human PDZD8-T527D or PDZD8-T527E were generated as described above in a), except that PDZD8-T527D or PDZD8-T527E were introduced to the N2 strain. The *aak-2*$^{-/-}$ nematode strains expressing human PDZD8, PDZD8-T527D, or PDZD8-T527E were generated similarly, but using *aak-2*$^{-/-}$ nematode as the background strain. Strains expressing PDZD8, PDZD8-T527A, PDZD8-T527D, and PDZD8-T527E were determined to express the various proteins at similar levels by immunoblotting and qPCR (performed as described above, but using the primers 5'-AAGACCCGCTGATCGACTTC-3' and 5'-GTGTGCTTGCGCTTGATGAT-3' to quantify the mRNA levels of PDZD8), were chosen for further experiments. For all nematode experiments, worms at the L4 stage were used, except those for CR 3 days after L4.

Cell lines and viruses

In this study, no cell line used is on the list of known misidentified cell lines maintained by the International Cell Line Authentication Committee (https://iclac.org/databases/cross-contaminations/). HEK293T cells (cat. CRL-3216) were purchased from ATCC and were authenticated by STR sequencing. *PDZD8*$^{-/-}$ MEFs[1] and *AMPKα*$^{-/-}$ HEK293T cells[6] were generated and validated as described previously. Cells were maintained in Dulbecco's modified Eagle's medium (DMEM) supplemented with 10% fetal bovine serum (FBS), 100 IU penicillin, and 100 mg/mL streptomycin at 37 °C in a humidified incubator containing 5% CO_2. Cells were verified to be free of mycoplasma contamination. PEI at a final concentration of 10 μM was used to transfect cells (for ectopic expression). The total DNA to be transfected for each plate was adjusted to the same amount by using the relevant empty vector. Transfected cells were harvested at 24 h after transfection.

Lentiviruses for stable expression (expressed at close-to-endogenous levels) were packaged in HEK293T cells (in DMEM supplemented with 10% FBS and MEM non-essential amino acids; approximately 2 mL) by transfection using Lipofectamine 2000. At 30 h post-transfection, the medium was collected and centrifuged at 5,000× *g* for 3 min at room temperature. The supernatant was mixed with 10 μg/mL polybrene, and was added to MEFs, followed by centrifugation at 3,000× *g* for 30 min at room temperature (spinfection). Cells were incubated for another 24 h before further

treatments, and the expression levels of stably expressed protein were determined by immunoblotting. In particular, for determining the expression levels of nematode pdzd-8 and its S536A mutant in MEFs, qPCR was performed with the following primers: 5'-CGAACACCGAATCTGTTGCC-3' and 5'- TTGAGGCACTCGAGCACTTT-3' for *pdzd-8*, and 5'-GACTTCAACAGCAACTCCCAC-3' and 5'-TCCACCACCCTGTTGCTGTA-3' for mouse *Gapdh*.

For glucose starvation, cells were rinsed twice with PBS, then incubated in glucose-free DMEM supplemented with 10% FBS and 1 mM sodium pyruvate for desired periods at 37 °C.

Data reporting

The chosen sample sizes were similar to those used in this field: n = 3-10 samples to evaluate the levels of metabolites in tissues[7-11] and nematodes[12-14]; n = 3-9 samples to determine OCR in tissues[11,15] and nematodes[16-18], and n = 6-10 samples to determine mitochondrial ROS in tissues[19] and nematodes[20,21]; n = 3-4 samples to determine the expression levels and phosphorylation levels of a specific protein in animal cells or tissues[22]; n = 3-4 samples to determine the mRNA levels of a particular gene[22]; n = 200 worms were used to determine lifespan[23-25]; and n = 60 worms were used to determine healthspan[26-28]. No statistical methods were used to predetermine the sample size. All experimental findings were repeated as stated in figure legends, and all additional replication attempts were successful. For animal experiments, mice or nematodes were

maintained under the same condition or place. For cell experiments, cells of each genotype were cultured in the same CO_2 incubator and were parallel-seeded and randomly assigned to different treatments. Each experiment was designed and performed along with controls, and samples for comparison were collected and analyzed under the same conditions. Randomization was applied wherever possible. For example, during MS analyses for metabolites, samples were processed and applied to the mass spectrometer in random orders. For animal experiments, sex-matched (only for mice) and age-matched litter-mate animals in each genotype were randomly assigned to different treatments. Otherwise, randomization was not performed. For example, when performing immunoblotting, samples needed to be loaded in a specific order to generate the final figures. Blinding was applied wherever possible. For example, samples, cages, or agar plates during sample collection and processing were labeled as code names that were later revealed by the individual who picked and treated animals or cells but did not participate in sample collection and processing until assessing the outcome. Similarly, during microscopy data collection and statistical analysis, the fields of view were chosen on a random basis, and were performed by different operators, preventing potentially biased selection for desired phenotypes.

CR treatments of mice

Unless stated otherwise, mice were housed with free access to water and standard diet (65% carbohydrate, 11% fat, 24% protein) under specific pathogen-free conditions. The light was on from 8:00 to 20:00, with the temperature kept at 21-24 °C and humidity at

40-70%. Only male mice were used in the study, and male littermate controls were used throughout the study. For CR, mice were individually caged for 1 month before treatment; each mouse was fed 2.5 g of standard diet (70% of ad libitum food intake for a mouse at 4 months old and above) at 5 p.m. each day;

Determination of mouse running capacity and grip strength

The maximal running capacity was determined as described previously[29], with minor modifications. Briefly, mice were trained on Rodent Treadmill NG (UGO Basile, cat. 47300) at 10 m/min for 5 min for 2 days with a normal light-dark cycle, and tests were performed during the dark period. Before the experiment, mice were fasted for 2 h. The treadmill was set at a 15° incline, and the speed of the treadmill was set to increase in a ramp mode (10 m/min for 10 min followed by an increase to a final speed of 18 m/min within 15 min). Mice were considered exhausted and removed from the treadmill, following the accumulation of 5 or more shocks (0.1 mA) per minute for two consecutive minutes. The distances traveled were recorded as the running capacity.

Grip strength was determined on a grip strength meter (Ugo Basile, cat. 47200) following the protocol described previously[28]. Briefly, the mouse was held by its tail and lowered ("landed") until all four limbs grasped the T-bar connected to a digital force gauge. The mouse was further lowered to the extent that the body was horizontal to the apparatus and then slowly, steadily drawn away from the T-bar until all four limbs were removed from the bar, which gave rise to the peak force in grams. Each mouse

was repeated 5 times with 5-min intervals between measurements.

Determination of body composition

Lean and fat body mass were measured by quantitative magnetic resonance (EchoMRI -100H Analyzer; Echo Medical Systems) as described previously[11]. Briefly, the system was pre-calibrated with oil standard. Mice were individually weighed, inserted into a restrainer tube, and immobilized by gently inserting a plunger. The mouse was then positioned to a gesture that curled up like a donut, with its head against the end of the tube. The body composition of each mouse was measured with two repeated runs, and the average values were taken for further analysis.

Determination of energy expenditure

Mouse EE was determined by a metabolic cage system (Promethion Line, CAB-16-1-EU; Sable Systems International) as described previously[30]. Briefly, the system was maintained in a condition identical to that for housing mice. Each metabolic cage in the 16-cage system consisted of a cage with standard bedding, a food hopper, and a water bottle connected to load cells for continuous monitoring. To minimize the stress of a new environment, mice were acclimatized for 1 week before data collection. Mice subjected to CR or ad libitum fed were randomly assigned/housed to prevent systematic errors in measurement. Body weights and fat proportion of mice were determined before and after the acclimation and the food and water intake daily. Mice found not acclimatized to the metabolic cage (for example, resistance to eating and drinking) were

removed from the study. Data acquisition (5-min intervals for each cage) and instrument control were performed using MetaScreen software (v.2.3.15.12, Sable Systems), and raw data were processed using Macro Interpreter (v.2.32, Sable Systems). Ambulatory activity and position were monitored using XYZ beam arrays with a beam spacing of 0.25 cm (beam breaks), and the mouse pedestrial locomotion (walking distance) within the cage was calculated accordingly. Respiratory gases were measured using the GA-3 gas analyzer (Sable Systems) equipped with a pull-mode, negative-pressure system. Airflow was measured and controlled by FR-8 (Sable Systems), with a set flow rate of 2,000 mL min^{-1}. Oxygen consumption (VO_2) and carbon dioxide production (VCO_2) were reported in mL per minute. Water vapor was measured continuously, and its dilution effect on O_2 and CO_2 was compensated mathematically in the analysis stream. EE was calculated using the Weir equation: kcal $h^{-1} = 60 \times (0.003941 \times VO_2 + 0.001106 \times VCO_2$. Differences in average EE were analyzed by ANCOVA using body weight as the covariate. The RQ was calculated as VCO_2/VO_2.

Histology

Muscle fiber types were determined as described previously[31,32]. Briefly, mice were sacrificed by cervical dislocation, and the muscle tissues were quickly excised, followed by frozen in the isopentane (pre-chilled in liquid nitrogen) for 2 min (until they appeared chalky white). The tissues were then quickly transferred to the embedding molds containing O.C.T. Compound, and were frozen in liquid nitrogen for

another 10 min. The embedded tissues were then sectioned into 6-μm slices at -20 °C using a CM1950 Cryostat (Leica), followed by fixing in 4% paraformaldehyde for 10 min, and were then washed with PBS for 5 min at room temperature. After incubating with PBST (PBS supplemented with 5% (v/v) Triton X-100) for 10 min, the sections were blocked with BSA Solution (PBS containing 5% (m/v) BSA) for 30 min at room temperature. Muscle fibers were stained with the antibody against MHCIIb (6 μg/mL, diluted in BSA Solution) overnight at 4 °C, followed by washing with PBS 3 times, 5 min each at room temperature. The sections were then incubated with Alexa Fluor 488-conjugated, goat anti-mouse IgM antibody (1:200 diluted in BSA Solution) for 1 h at room temperature in a dark humidified chamber, followed by washing with PBS for 3 times, 5 min each, incubated with 4% paraformaldehyde for 2 min, and then washed with PBS twice, 5 min each, all at room temperature. The sections were then incubated with antibody against MHCI (6 μg/mL, diluted in BSA Solution) for 3 h at room temperature in a dark humidified chamber, followed by washing with PBS buffer 3 times, 5 min each at room temperature, and then incubated with Alexa Fluor 594-conjugated, goat anti-mouse IgG2b antibody (1:200 diluted in BSA Solution) for another 1 h at room temperature in a dark humidified chamber, followed by washing with PBS buffer for 3 times, 5 min each at room temperature. After fixing with 4% paraformaldehyde for 2 min and washing with PBS twice, 5 min each at room temperature, the sections were incubated with the antibody against MHCIIa (6 μg/mL, diluted in BSA Solution) for 3 h at room temperature in a dark humidified chamber, followed by washing with PBS buffer for 3 times, 5 min each at room temperature, and

then incubated in Alexa Fluor 647-conjugated goat anti-mouse IgG1 antibody (1:200 diluted in BSA Solution) for another 1 h at room temperature in a dark humidified chamber, followed by washing with PBS buffer for 3 times, 5 min each at room temperature. Tissue sections were mounted with 90% glycerol and visualized on a Zeiss LSM980 microscope.

Evaluation of nematode lifespan and healthspan

To determine the lifespan of nematodes, the synchronized worms were cultured to the L4 stage before being transferred to the desired agar plates for determining lifespan. For 2-DG or NAC treatment, 4 mM 2-DG or 4 mM NAC was freshly dissolved in water and added to warm NGM supplemented with 1.7% (w/v) agar before pouring to make the NGM plates. The plates were stored at 20 °C. For CR, OP50 bacteria were diluted to the concentration of 10^9/mL (along with 10^{12}/mL as the control, ad libitum fed group; see ref. [33]). The diluted bacteria were isopycnically spread on the NGM plates (for a 35-mm NGM plate, 250 μL of bacteria were used) containing 50 mg/L ampicillin and 50 mg/L kanamycin. Worms were transferred to new plates every 2 d. Live and dead worms were counted during the transfer. Worms that displayed no movement upon gentle touching with a platinum picker were judged dead. Kaplan-Meier curves were graphed by Prism 9 (GraphPad Software), and the statistical analysis data was analyzed using SPSS 27.0 (IBM).

Pharyngeal pumping rates, assessed as the numbers of contraction-relaxation cycles of

the terminal bulb on the nematode pharynx within 1 min, were determined as described previously[34], with minor modification. Briefly, worms were treated with 2-DG or subjected to CR for 2 days, followed by being picked and placed on a new NGM plate containing *E. coli*. After 10 min of incubation at room temperature, the contraction-relaxation cycles of the terminal bulb of each worm were recorded on a stereomicroscope (M165 FC, Leica) through a 63× objective for a consecutive 4 min using the Capture software (v.2021.1.13, Capture Visualization), and the average contraction-relaxation cycles per min were calculated using the Aimersoft Video Editor software (v.3.6.2.0, Aimersoft).

The resistance of nematodes to oxidative stress was determined as described previously[26]. Briefly, worms were treated with 2-DG or subjected to CR for 2 days. Some 20 worms were then transferred to an NGM plate containing 15 mM $FeSO_4$. Worms were then cultured at 20 °C on such a plate, during which the live and dead worms were counted every 1 h.

Determination of mRNA levels of antioxidative genes in nematodes

Levels of antioxidative gene expression were determined through the RNA-sequencing performed by Seqhealth Technology Co., Ltd. (Wuhan, China). Briefly, RNAs from approximately 1,000 worms (treated with 2-DG, or undergone CR) were extracted as described in the section of determining the knockdown efficiency of *glna-2*. The residual DNA in each sample was removed by treating with RNase-free DNase I for 30

min at 37 °C, and the quality of RNA was double-checked through agarose gel (1.5%) electrophoresis and the NanoDrop OneC Microvolume UV-Vis Spectrophotometer (Thermo), followed by quantified on a Qubit 3 Fluorometer after staining with the Qubit RNA BR kit. Some 2 μg of total RNAs were then subjected to construct cDNA libraries using the Collibri Stranded RNA Library Prep Kit for Illumina Systems, following the manufacturer's instructions. The cDNAs in the library with a length of 200-500 bps were enriched using KAPA HyperPure magnetic beads following the manufacturer's instructions, followed by quantification using the Collibri Library Quantification Kit, and sequenced on a DNBSEQ-500 sequencer (MGI Tech Co., Ltd.) under the PEI150 mode. The low-quality sequences, including a) reads containing more than 50% bases with quality lower than 20 in a sequence; b) reads with more than 5% bases unknown; and c) reads containing adaptor sequences were removed from the total reads using the Trimmomatic (version 0.36) software as described previously[35].

Expression levels of the antioxidative gene were quantified through their RPKM (reads per kilobase of transcript per million reads mapped) values. To acquire the RPKM value of each gene, reads were first mapped to the reference sequence of *C. elegans* using the STAR software (version 2.5.3a) as described previously[36] to make sure that reads could be uniquely mapped to the gene chosen to calculate the RPKM values. For genes with more than one alternative transcript, the longest transcript was selected to calculate the RPKM. The RPKM was calculated by the featureCounts software (version 1.5.1) as described previously[37]. RPKM values for each antioxidative gene were plotted using

Prism 9 (GraphPad) software.

In particular, to determine the mRNA levels of *sod-4* in nematodes, RT-PCR was used. Some 1,000 worms were collected with 15 mL of M9 buffer containing 0.05% Triton X-100 (v/v), followed by centrifugation for 2 min at 1,000× *g*. The sediment was then washed with 1 mL of M9 buffer twice and then lysed with 1 mL of TRIzol. Worms were then frozen in liquid nitrogen, thawed at room temperature, and then repeated freeze-thaw for another 2 times. The worm lysates were then placed at room temperature for 5 min, then mixed with 0.2 mL of chloroform, followed by vigorous shaking for 15 s. After centrifugation at 12,000× *g* for 15 min at 4 °C, 450 μL of each upper aqueous layer was transferred to an RNase-free tube. The RNA was then precipitated by adding 450 μL of isopropanol, followed with centrifugation at 12,000× *g* for 30 min at 4 °C. The pellet was washed twice with 75% ethanol, once with 100% ethanol, and dissolved with 20 μL of DEPC-treated water. The concentration of RNA was determined by a NanoDrop 2000 spectrophotometer (Thermo). Some 1 μg of RNA was diluted with DEPC-treated water to a final volume of 10 μL, heated at 65 °C for 5 min, and chilled on ice immediately. The Random Primer Mix, Enzyme Mix and 5× RT buffer (all from the ReverTra Ace qPCR RT Kit) were then added to the RNA solution, followed by incubation at 37 °C for 15 min, and then at 98 °C for 5 min on a thermocycler. The reverse-transcribed cDNA was quantified with Maxima SYBR Green/ROX qPCR Master Mix on a LightCycler 480 II System (Roche) with the following programs: pre-denaturing at 95 °C for 10 min; denaturing at 95 °C for 10 s, then annealing and

extending at 65 °C for 30 s in each cycle [determined according to the amplification curves, melting curves, and bands on agarose gel of serial pilot reactions (in which a serial annealing temperature was set according to the estimated annealing temperature of each primer pair) of each primer pair, and same hereafter], for a total of 45 cycles. Primer sequences are as follows: *C. elegans ama-1*, 5'-GACATTTGGCACTGCTTTGT-3' and 5'-ACGATTGATTCCATGTCTCG-3'; *C. elegans sod-4*, 5'-CGGCTTCCGGAGACACATTA-3' and 5'-ACCACACTTCGGCCAATGAT -3'.

Plasmids

Full-length cDNAs used in this study were obtained either by PCR using cDNA from MEFs or nematodes, or by purchasing from Origene or Sino Biological. Mutations of PDZD8, GLS1, and pdzd-8 were performed by PCR-based site-directed mutagenesis using PrimeSTAR HS polymerase. Expression plasmids for various epitope-tagged proteins were constructed in the pcDNA3.3 vector (#K830001, Thermo) for transfection (ectopic expression) in HEK293T cells, or in the pBOBI vector for lentivirus packaging (stable expression) in HEK293T cells. PCR products were verified by sequencing (Invitrogen, China). *Escherichia coli* strain DH5α (cat. #PTA-1977) was purchased from ATCC and was used to amplify plasmids. All plasmids used in this study were purified using the CsCl density gradient ultracentrifugation method. The expression plasmids constructed in this study have been deposited to Addgene (https://www.addgene.org/Sheng-cai_Lin/).

IP and IB assays

To verify the phosphorylation of nematode pdzd-8 by AMPK (using the anti-pan-phospho-AMPK substrate antibody), a 10 cm-dish of HEK293T cells was transfected with Myc-tagged pdzd-8-expression plasmids. Cells were lysed with 1 mL of ice-cold Triton lysis buffer (20 mM Tris-HCl, pH 7.5, 150 mM NaCl, 1 mM EDTA, 1 mM EGTA, 1% (v/v) Triton X-100, 2.5 mM sodium pyrophosphate, 1 mM β-glycerophosphate, with protease inhibitor cocktail), followed by sonication and centrifugation at 4 °C for 15 min. Cell lysates were incubated with anti-Myc-tag (1:100) antibodies, along with protein A/G beads (1:100 dilution, balanced with Triton lysis buffer), added into the supernatant, and mixed for 15 min at 4 °C. The beads were washed with 200 times volume of ice-cold Triton lysis buffer 3 times at 4 °C, mixed with an equal volume of 2× SDS sample buffer, and boiled for 10 min before immunoblotting.

To analyze the levels of p-PDZD8 muscle tissues, mice were anesthetized after indicated treatments. Freshly excised (or freeze-clamped) tissues were immediately lysed with ice-cold Triton lysis buffer (5 μL/mg tissue weight), followed by homogenized and centrifuged at 20,000× *g* for 10 min at 4 °C. The supernatant was then mixed with an equal volume of 2× SDS sample buffer, followed by boiling for 10 min, and then directly subjected to immunoblotting. To analyze the levels of PDZD8 in nematodes, about 150 nematodes cultured on the NGM plate were collected for each sample. Worms were quickly washed with ice-cold M9 buffer containing Triton X-100,

and were lysed with 150 μL of ice-cold Triton lysis buffer. The lysates were mixed with 5× SDS sample buffer, followed by homogenization and centrifugation as described above, and then boiled before being subjected to immunoblotting. All samples were subjected to immunoblotting on the same day of preparation, and any freeze-thaw cycles were avoided.

For immunoblotting, the SDS-polyacrylamide gels were prepared in-house. The thickness of the gels used in this study was 1.0 mm. Samples of less than 10 μL were loaded into wells, and the electrophoresis was run at 100 V (by PowerPac HC High-Current Power Supply, Bio-Rad) in a Mini-PROTEAN Tetra Electrophoresis Cell (Bio-Rad). In this study, all samples were resolved on 8% resolving gels, except the deletion mutants of pdzd-8 (aa 1-256, 257-416, 870-1047, and 1048-1365) on 15%. The resolved proteins were then transferred to the PVDF membrane (0.45 μm, cat. IPVH00010, Merck). The PVDF membrane was then blocked by 5% (w/v) BSA (for all antibodies against phosphorylated proteins) or 5% (w/v) non-fat milk (for all antibodies against total proteins) dissolved in TBST for 2 h on an orbital shaker at 60 rpm at room temperature, followed by rinsing with TBST for twice, 5 min each. The PVDF membrane was then incubated with the desired primary antibody overnight at 4 °C on an orbital shaker at 60 rpm, followed by rinsing with TBST three times, 5 min each at room temperature, and then the secondary antibodies for 3 h at room temperature with gentle shaking. The secondary antibody was then removed, and the PVDF membrane was further washed with TBST 3 times, 5 min each, at room

temperature. PVDF membrane was incubated in an ECL mixture (by mixing equal volumes of ECL solution and Peroxide solution for 5 min), then life with Medical X-Ray Film (FUJIFILM). The films were then developed with X-OMAT MX Developer (Carestream) and X-OMAT MX Fixer and Replenisher solutions (Carestream) on a Medical X-Ray Processor (Carestream) using Developer (Model 002, Carestream). For re-probing, the PVDF membrane was boiled in water for 5 min, followed by washing with TBST 3 times, 5 min each, at room temperature, and then incubated with desired primary and secondary antibodies. The developed films were scanned using a Perfection V850 Pro scanner (Epson) with an Epson Scan software (v.3.9.3.4), and were cropped using Photoshop 2023 software (Adobe). Levels of total proteins and phosphorylated proteins were analyzed on separate gels, and representative immunoblots are shown. Uncropped immunoblots are uploaded as a "Full scans" file

Determination of rates of glutaminolysis

To determine the glutaminolysis rates in MEFs, cells from one 10-cm dish (60–70% confluence) were collected for each measurement. MEFs were glucose-starved for desired periods of time by incubating with triple-free (free of glucose, pyruvate, and glutamine) DMEM supplemented with 4 mM glutamine, 1 mM sodium pyruvate, 100 μM PA, 1 mM carnitine (according to ref. [38]), and 10% FBS. At 20 min before sample collection, cells were incubated with pre-warmed triple-free DMEM supplemented with 3 mM glutamine, 1 mM [U-^{13}C]-glutamine, 1 mM sodium pyruvate, 100 μM PA, 1 mM carnitine, and 10% FBS. To determine the rates of glutaminolysis in *C. elegans*, 1,000

nematodes were incubated with 8 mM [U-^{13}C]-glutamine (final concentration, added to a 6-cm NGM plate containing OP50 bacteria) for 24 h, followed by washing and collecting with M9 buffer. Cells and worms were then lysed with 1 mL of 80% methanol (v/v in water) containing 10 μg/mL myristic-d27 acid as an internal standard (IS), followed by 20 s of vortex. After centrifugation at 15,000× *g* for 15 min at 4 °C, 600 μL of each supernatant (aqueous phase) was freeze-dried in a vacuum concentrator (a LABCONCO #7310037 centrifuge connected to a LABCONCO #7460037 cold trap and an EDWARDS nXDS15i pump) at 4 °C for 24 h. The lyophilized samples were then subjected to derivatization by vortexing for 1 min after mixing each with 50 μL of freshly prepared methoxyamine hydrochloride (20 mg/mL in pyridine), followed by incubation at 4 °C for 1 h. The mixtures were sonicated at 0 °C by bathing in an ice slurry for 10 min, and were then incubated at 37 °C for 1.5 h, followed by mixing with 50 μL of MTBSTFA and incubated at 55 °C for 1 h. Before subjecting to GC-MS, samples were centrifuged at 15,000× *g* for 10 min, and some 60 μL of each supernatant was loaded into an injection vial (cat. 5182-0714, Agilent; with an insert (cat. HM-1270, Zhejiang Hamag Technology)) equipped with a snap cap (cat. HM-0722, Zhejiang Hamag Technology). GC was performed on an HP-5MS column (30 m × 0.25 mm i.d., 0.25 μm film thickness; cat. 19091S-433; Agilent) using a GC/MSD instrument (7890-5977B, Agilent) as described previously[11]. Briefly, the injector temperature of GC/MSD was set at 260 °C. The column oven temperature was first held at 70 °C for 2 min, then increased to 180 °C at the rate of 7 °C/min, then to 250 °C at 5 °C/min, then to 310 °C at 25 °C/min, where it was held for 15 min. The MSD transfer temperature was 280 °C.

The MS quadrupole and source temperature were maintained at 150 °C and 230 °C, respectively. Measurements were performed in both a scan mode (to assure the quality and purity of each TCA cycle intermediate peak) and a selected ion monitoring (SIM) mode (to maximize the sensitivity of GC-MS for quantifying each metabolite/isotopomer). In SIM mode, the fragment ion with m/z values of [M-57] (where M is the molecular mass of each derivatized metabolite, and the loss of the 57-Da facile is attributed to the loss of the *tert*-butyl moiety of the metabolite in the GC of each compound) was set as the quantitative ion. To ensure that all possible isotopomer peaks, including those of naturally occurring isotopes of a specific metabolite (with n carbon atoms), were recorded, the m/z values ranging from [M-57] to [M-57] + n + 1 were included during the data collection. In particular, for pyruvate and α-KG, m/z values from [M-57] to [M-57] + n + 2 were recorded, owing to the oximation of these two metabolites during the derivatization. The following m/z values were used for each compound: 174, 175, 176, 177 and 178 for pyruvate; 289, 290, 291, 292 and 293 for succinate; 287, 288, 289, 291 and 292 for fumarate; 346, 347, 348, 349, 350, 351 and 352 for α-KG; 419, 420, 421, 422 and 423 for malate; 418, 419, 420, 421 and 422 for aspartate; 432, 433, 434, 435, 436 and 437 for glutamate; 431, 432, 433, 434, 435, 436 for glutamine; and 591, 592, 593, 594, 595, 596 and 597 for citrate. Data were collected using the MassHunter GC/MS Acquisition software (v.B.07.04.2260, Agilent). For quantification, peaks were extracted and integrated using GC-MS MassHunter Workstation Qualitative Analysis software (v.B.07.01SP1, Agilent), and were corrected for naturally occurring isotopes using the IsoCor software[39,40] with the matrix-based

method.

Determination of NAD$^+$

To determine levels of NAD$^+$, high-performance liquid chromatography-mass spectrometry (HPLC-MS) was performed[11]. Briefly, some 50 mg of fleshly excised (using a freeze-clamp) muscle tissue was immediately frozen in liquid nitrogen, and homogenized in 1 mL of ice-cold methanol. The lysates were then mixed with 1 mL of chloroform and 400 μL of water (containing 4 μg/mL [U-^{13}C]-glutamine as an IS), followed by 20 s of vortexing. After centrifugation at 15,000× *g* for another 15 min at 4 °C, 800 μL of the aqueous phase was collected, lyophilized in a vacuum concentrator at 4 °C for 8 h, and then dissolved in 30 μL of 50% (v/v, in water) acetonitrile, followed by loading 20 μL of solution into an injection vial (cat. 5182-0714, Agilent; with an insert (cat. HM-1270, Zhejiang Hamag Technology)) equipped with a snap cap (cat. HM-2076, Zhejiang Hamag Technology). Measurements were based on ref. [41] using a QTRAP MS (QTRAP 5500, SCIEX) interfaced with a UPLC system (ExionLC AD, SCIEX). Some 2 μL of samples were loaded onto a HILIC column (ZIC-pHILIC, 5 μm, 2.1 × 100 mm, PN: 1.50462.0001, Millipore). The mobile phase consisted of 15 mM ammonium acetate containing 3 mL/L ammonium hydroxide (>28%, v/v) in the LC-MS grade water (mobile phase A) and LC-MS grade, 90% (v/v) acetonitrile in LC-MS grade water (mobile phase B) run at a flow rate of 0.2 mL/min. Metabolites were separated with the following HPLC gradient elution program: 95% B held for 2 min, then 45% B in 13 min, held for 3 min, and then back to 95% B for 4 min. The mass

spectrometer was run on a Turbo V ion source in negative mode with a spray voltage of -4,500 V. Source temperature was set at 550 °C, Gas No.1 at 50 psi, Gas No.2 at 55 psi, and curtain gas at 40 psi. Metabolites were measured using the multiple reactions monitoring mode (MRM), and declustering potentials and collision energies were optimized using analytical standards. The following transitions (parent ion/daughter ion) were used for monitoring each compound: 662.0/540.1 for NAD^+ and 149.9/114 for [U-^{13}C]-glutamine. Data were collected using Analyst software (v.1.7.1, SCIEX), and the relative amounts of metabolites were analyzed using MultiQuant software (v.3.0.3, SCIEX).

Measurements of adenylates

Levels of AMP, ADP, and ATP were analyzed by capillary electrophoresis-based mass spectrometry (CE-MS) as described previously[1], with minor modifications. Briefly, each measurement required 100 mg of muscle tissues. Muscle tissues were quickly excised by freeze-clamping from anesthetized mice, followed by grinding in 1 mL of methanol containing IS1 (50 µM L-methionine sulfone, 50 µM D-campher-10-sulfonic acid, dissolved in water; 1:500 (v/v) added to the methanol and used to standardize the metabolite intensity and to adjust the migration time). The lysate was then mixed with 1 mL of chloroform and 400 µL of water, followed by 20 s of vortexing. After centrifugation at 15,000× *g* for 15 min at 4 °C, 450 µL of aqueous phase was collected and was then filtrated through a 5-kDa cutoff filter (cat. OD003C34, PALL) by centrifuging at 12,000× *g* for 3 h at 4 °C. In parallel, quality control samples were

prepared by combining 10 µL of the aqueous phase from each sample and then filtered alongside the samples. The filtered aqueous phase was then freeze-dried in a vacuum concentrator at 4 °C, and then dissolved in 100 µL of water containing IS2 (50 µM 3-aminopyrrolidine dihydrochloride, 50 µM N,N-diethyl-2-phenylacetamide, 50 µM trimesic acid, 50 µM 2-naphtol-3,6-disulfonic acid disodium salt, dissolved in methanol; used to adjust the migration time). A total of 20 µL of re-dissolved solution was then loaded into an injection vial (cat. 9301-0978, Agilent; equipped with a snap cap (cat. 5042-6491, Agilent)). Before CE-MS analysis, the fused-silica capillary (cat. TSP050375, i.d. 50 µm × 80 cm; Polymicro Technologies) was installed in a CE/MS cassette (cat. G1603A, Agilent) on the CE system (Agilent Technologies 7100). The capillary was then pre-conditioned with Conditioning Buffer (25 mM ammonium acetate, 75 mM diammonium hydrogen phosphate, pH 8.5) for 30 min, followed by balanced with Running Buffer (50 mM ammonium acetate, pH 8.5; freshly prepared) for another 1 h. CE-MS analysis was run at anion mode, during which the capillary was washed by Conditioning Buffer, followed by injection of the samples at a pressure of 50 mbar for 25 s, and then separation with a constant voltage at -30 kV for another 40 min. Sheath Liquid (0.1 µM hexakis(1H, 1H, 3H-tetrafluoropropoxy)phosphazine, 10 µM ammonium trifluoroacetate, dissolved in methanol/water (50% v/v); freshly prepared) was flowed at 1 mL/min through a 1:100 flow splitter (Agilent Technologies 1260 Infinity II; actual flow rate to the MS: 10 µL/min) throughout each run. The parameters of the mass spectrometer (Agilent Technologies 6545) were set as: a) ion source: Dual AJS ESI; b) polarity: negative; c) nozzle voltage: 2,000 V; d) fragmentor

voltage: 110 V; e) skimmer voltage: 50 V; f) OCT RFV: 500 V; g) drying gas (N_2) flow rate: 7 L/min; h) drying gas (N_2) temperature: 300 °C; i) nebulizer gas pressure: 8 psig; j) sheath gas temperature: 125 °C; k) sheath gas (N_2) flow rate: 4 L/min; l) capillary voltage (applied onto the sprayer): 3,500 V; m) reference (lock) masses: m/z 1,033.988109 for hexakis(1H, 1H, 3H-tetrafluoropropoxy)phosphazine, and m/z 112.985587 for trifluoroacetic acid; n) scanning range: 50-1,100 m/z; and n) scanning rate: 1.5 spectra/s. Data were collected using MassHunter LC/MS acquisition 10.1.48 (Agilent), and were processed using Qualitative Analysis B.06.00 (Agilent). The peak areas of adenylates were calculated using the following parameters (m/z, retention time (min)): a) AMP: 346.0558, 9.302; b) ADP: 426.0221, 10.930; and c) ATP: 505.9885, 11.848. Note that the retention time of each adenylate may vary between each run and can be adjusted by isotope-labeled standards (dissolved in individual cell or tissue lysates) run between each sample, so do IS1 and IS2.

Determination of oxygen consumption rates

The oxygen consumption rates (OCR) of MEFs were measured as described previously[1]. Briefly, cells were plated at 10,000 cells per well on a 96-well Seahorse XF Cell Culture Microplate (Agilent) in full medium (DMEM containing 10% FBS) overnight before the experiment, followed by glucose starvation for desired time periods. Medium was then changed to Seahorse XF Base Medium supplemented with 10% FBS, 25 mM glucose (not included under starvation condition, and same hereafter), 4 mM glutamine (GlutaMAX) and 1 mM sodium pyruvate 1 h before the experiment.

Cells were then placed in a CO_2-free, XF96 Extracellular Flux Analyzer Prep Station (Agilent) at 37 °C for 1 h. OCR was then measured at 37 °C in an XF96 Extracellular Flux Analyzer (Agilent), with a Seahorse XFe96 sensor cartridge (Agilent) pre-equilibrated in Seahorse XF Calibrant solution in a CO_2-free incubator at 37 °C overnight. The assay was performed on a Seahorse XFe96 Analyzer (Agilent) at 37 °C following the manufacturer's instruction. Concentrations of respiratory chain inhibitors used during the assay were: oligomycin A at 10 μM, FCCP at 10 μM, antimycin A at 1 μM and rotenone at 1 μM (all final concentrations). Data were collected using Wave 2.6.1 Desktop software (Agilent) and exported to Prism 9 (GraphPad) for further analysis according to the manufacturer's instructions.

The OCR of nematodes was measured as described previously[42]. Briefly, nematodes were washed with M9 buffer for 3 times. Some 15 to 25 nematodes were then suspended in 200 μL of M9 buffer, and were added to a well on a 96-well Seahorse XF Cell Culture Microplate. The medium was then changed to Seahorse XF Base Medium supplemented with 10% FBS, 25 mM glucose (not included under starvation condition, and same hereafter), 4 mM glutamine (GlutaMAX), and 1 mM sodium pyruvate 1 h before the experiment. Worms were then placed in a CO_2-free, XF96 Extracellular Flux Analyzer Prep Station (Agilent) at 20 °C for 1 h. OCR was then measured at 20 °C in an XF96 Extracellular Flux Analyzer (Agilent), with a Seahorse XFe96 sensor cartridge (Agilent) pre-equilibrated in Seahorse XF Calibrant solution in a CO_2-free incubator at 20 °C overnight. The assay was performed on a Seahorse XFe96 Analyzer (Agilent) at

20 °C following the manufacturer's instruction. Concentrations of respiratory chain inhibitors used during the assay were 10 μM FCCP and 40 mM sodium azide (final concentrations). Data were collected using Wave 2.6.1 Desktop software (Agilent) and exported to Prism 9 (GraphPad) for further analysis according to the manufacturer's instructions. At the end of the assay, the exact number of nematodes in each well was determined on a Cell Imaging Multi-Mode Reader (Cytation 1, BioTek) and was used for normalizing/correcting OCR results.

Determination of mitochondrial ROS

For detecting the mitochondrial ROS levels in MEFs, cells were grown in 35-mm glass-bottom dishes (cat. D35-20-10-N, In Vitro Scientific) to 50% confluence. Cells were treated with 5 μM (final concentration) MitoSOX dye for 0.5 h at 37 °C, then washed three times with 2 mL of pre-warmed culture medium, and incubated in fresh medium containing ProLong™ Live Antifade Reagent before imaging. During imaging, live cells were kept at 37 °C, 5% CO_2, in a humidified incubation chamber (Incubator PM S1, Zeiss). Images were taken using an LSM 980 (Zeiss) with a 63× 1.4 NA oil objective, during which a DPSS laser module (Lasos) at 594 nm was used to excite mitoSOX. The parameters, including 'PMT voltage', 'Offset', 'Pinhole' and 'Gain', were kept unchanged between each picture taken. The resolution of the image is 1,024×1,024 pixels. Images were processed and analyzed on Zen Blue 3.3 software (Zeiss), and formatted on Photoshop 2023 software (Adobe).

For detecting the mitochondrial ROS levels in nematodes, synchronized nematodes cultured to the L4 stage were treated with 2-DG or subjected to CR for 48 h. Nematodes were then treated with 5 μM (final concentration; added into the NGM plate containing the OP50 bacteria) MitoSOX dye for another 12 h at 20 °C, followed by placing on the center of an injection pad (prepared by placing 2 drops (approximately 50 μL) of boiling 4% agarose (w/v) onto the center of a glass coverslip (24 × 50 mm, 0.13–0.15 mm thickness), immediately followed by flattening with another coverslip, then dried at room temperature for 24 h). The pad was then subjected to imaging as described in those of MEFs, except that an LSM 900 (Zeiss) with a ×20, 0.8 NA plan-Apochromat air objective was used, during which a laser module URGB (cat. 400102-9301-000, Toptica) using a 10-mW laser line at 561 nm was applied. Images were processed by Zen 3.1 software (Zeiss), and formatted on Photoshop 2023 software (Adobe).

For detecting mitochondrial ROS levels in muscle tissues, mice were starved for desired time periods, and were sacrificed by cervical dislocation. The gastrocnemius muscle was then quickly excised and sliced to 0.05 cm^3 cubes, followed by immediately soaking in O.C.T. Compound at -20 °C for 20 min. The embedded tissues were then sectioned into 15-μm slices using a CM1950 Cryostat (Leica). Sections were stained with 40 mL of 5 μM (final concentration; by diluting the DMSO stock solution with PBS) MitoSOX dye for 30 min at 37 °C in a Coplin jar, followed by washing for 3 times, 5 min each with 40 mL of PBS at room temperature. Sections were then mounted with Antifade Mounting Medium, and were imaged on a DM4 B (Leica) microscope.

Statistical analysis

Statistical analyses were performed using Prism 9 (GraphPad Software), except for the survival curves, which were analyzed using SPSS 27.0 (IBM). Each group of data was subjected to the Kolmogorov-Smirnov test, Anderson-Darling test, D'Agostino-Pearson omnibus test, or Shapiro-Wilk test for normal distribution when applicable. An unpaired two-tailed Student's *t*-test was used to determine the significance between two groups of normally distributed data. Welch's correction was used for groups with unequal variances. An unpaired two-tailed Mann-Whitney test was used to determine significance between data without a normal distribution. For comparisons between multiple groups with one fixed factor, an ordinary one-way ANOVA was used, followed by Tukey, Sidak, Dunnett, or Dunn as specified in the legends. The assumptions of homogeneity of error variances were tested using F-test ($p > 0.05$). For comparison between multiple groups with two fixed factors, an ordinary two-way ANOVA was used, followed by Tukey's or Sidak's multiple comparisons test as specified in the legends. Geisser-Greenhouse's correction was used where applicable. The adjusted means and SEM, or SD, were recorded when the analysis met the above standards. Differences were considered significant when $p < 0.05$, or $p > 0.05$, with large differences of observed effects (as suggested in refs. [43,44]). Raw data and the statistical analysis data are also provided in this study as a "Source data" file.

References for Supplementary information

1 Li, M. *et al.* AMPK targets PDZD8 to trigger carbon source shift from glucose to glutamine. *Cell Res* (2024). https://doi.org:10.1038/s41422-024-00985-6

2 Brenner, S. The genetics of Caenorhabditis elegans. *Genetics* **77**, 71-94 (1974).

3 Mair, W. *et al.* Lifespan extension induced by AMPK and calcineurin is mediated by CRTC-1 and CREB. *Nature* **470**, 404-408 (2011). https://doi.org:10.1038/nature09706

4 Ma, T. *et al.* Low-dose metformin targets the lysosomal AMPK pathway through PEN2. *Nature* (2022). https://doi.org:10.1038/s41586-022-04431-8

5 Han, M. *et al.* A Systematic RNAi Screen Reveals a Novel Role of a Spindle Assembly Checkpoint Protein BuGZ in Synaptic Transmission in C. elegans. *Front Mol Neurosci* **10**, 141 (2017). https://doi.org:10.3389/fnmol.2017.00141

6 Li, M. *et al.* Hierarchical inhibition of mTORC1 by glucose starvation-triggered AXIN lysosomal translocation and by AMPK. *Life Metabolism* (2023). https://doi.org:10.1093/lifemeta/load005

7 Zhang, C. S. *et al.* Fructose-1,6-bisphosphate and aldolase mediate glucose sensing by AMPK. *Nature* **548**, 112-116 (2017). https://doi.org:10.1038/nature23275

8 Zong, Y. *et al.* Hierarchical activation of compartmentalized pools of AMPK depends on severity of nutrient or energy stress. *Cell Res* **29**, 460-473 (2019). https://doi.org:10.1038/s41422-019-0163-6

9 Hui, S. *et al.* Glucose feeds the TCA cycle via circulating lactate. *Nature* **551**, 115-118 (2017). https://doi.org:10.1038/nature24057

10 Hui, S. *et al.* Quantitative Fluxomics of Circulating Metabolites. *Cell metabolism* **32**, 676-688 e674 (2020). https://doi.org:10.1016/j.cmet.2020.07.013

11 Zhang, C. S. *et al.* The aldolase inhibitor aldometanib mimics glucose starvation to activate lysosomal AMPK. *Nat Metab* (2022). https://doi.org:10.1038/s42255-022-00640-7

12 Perez, C. L. & Van Gilst, M. R. A 13C isotope labeling strategy reveals the influence of insulin signaling on lipogenesis in C. elegans. *Cell metabolism* **8**, 266-274 (2008). https://doi.org:10.1016/j.cmet.2008.08.007

13 Falk, M. J. *et al.* Stable isotopic profiling of intermediary metabolic flux in developing and adult stage Caenorhabditis elegans. *J Vis Exp* (2011). https://doi.org:10.3791/2288

14 Vergano, S. S. *et al.* In vivo metabolic flux profiling with stable isotopes discriminates sites and quantifies effects of mitochondrial dysfunction in C. elegans. *Mol Genet Metab* **111**, 331-341 (2014). https://doi.org:10.1016/j.ymgme.2013.12.011

15 Schuh, R. A., Jackson, K. C., Khairallah, R. J., Ward, C. W. & Spangenburg, E. E. Measuring mitochondrial respiration in intact single muscle fibers. *Am J Physiol Regul Integr Comp Physiol* **302**, R712-719 (2012). https://doi.org:10.1152/ajpregu.00229.2011

16 Koopman, M. *et al.* A screening-based platform for the assessment of cellular respiration in Caenorhabditis elegans. *Nature protocols* **11**, 1798-1816 (2016).

https://doi.org:10.1038/nprot.2016.106

17 Sarasija, S. & Norman, K. R. Measurement of Oxygen Consumption Rates in Intact Caenorhabditis elegans. *J Vis Exp* (2019). https://doi.org:10.3791/59277

18 Ng, L. F. & Gruber, J. Measurement of Respiration Rate in Live Caenorhabditis elegans. *Bio Protoc* **9**, e3243 (2019). https://doi.org:10.21769/BioProtoc.3243

19 Vaccaro, A. *et al.* Sleep Loss Can Cause Death through Accumulation of Reactive Oxygen Species in the Gut. *Cell* **181**, 1307-1328 e1315 (2020). https://doi.org:10.1016/j.cell.2020.04.049

20 Dingley, S. *et al.* Mitochondrial respiratory chain dysfunction variably increases oxidant stress in Caenorhabditis elegans. *Mitochondrion* **10**, 125-136 (2010). https://doi.org:10.1016/j.mito.2009.11.003

21 Yang, W. & Hekimi, S. A mitochondrial superoxide signal triggers increased longevity in Caenorhabditis elegans. *PLoS Biol* **8**, e1000556 (2010). https://doi.org:10.1371/journal.pbio.1000556

22 Zhang, C. S. *et al.* The lysosomal v-ATPase-Ragulator complex Is a common activator for AMPK and mTORC1, acting as a switch between catabolism and anabolism. *Cell Metab.* **20**, 526-540 (2014). https://doi.org:10.1016/j.cmet.2014.06.014

23 Espada, L. *et al.* Loss of metabolic plasticity underlies metformin toxicity in aged Caenorhabditis elegans. *Nat Metab* **2**, 1316-1331 (2020). https://doi.org:10.1038/s42255-020-00307-1

24 Wu, L. *et al.* An Ancient, Unified Mechanism for Metformin Growth Inhibition

in C. elegans and Cancer. *Cell* **167**, 1705-1718 e1713 (2016). https://doi.org:10.1016/j.cell.2016.11.055

25 Martin-Montalvo, A. *et al.* Metformin improves healthspan and lifespan in mice. *Nat Commun* **4**, 2192 (2013). https://doi.org:10.1038/ncomms3192

26 De Rosa, M. J. *et al.* The flight response impairs cytoprotective mechanisms by activating the insulin pathway. *Nature* **573**, 135-138 (2019). https://doi.org:10.1038/s41586-019-1524-5

27 Yuan, J. *et al.* Two conserved epigenetic regulators prevent healthy ageing. *Nature* **579**, 118-122 (2020). https://doi.org:10.1038/s41586-020-2037-y

28 Zhang, H. *et al.* NAD(+) repletion improves mitochondrial and stem cell function and enhances life span in mice. *Science* **352**, 1436-1443 (2016). https://doi.org:10.1126/science.aaf2693

29 Park, S. J. *et al.* DNA-PK Promotes the Mitochondrial, Metabolic, and Physical Decline that Occurs During Aging. *Cell metabolism* **26**, 447 (2017). https://doi.org:10.1016/j.cmet.2017.07.005

30 Liu, P. *et al.* Blocking FSH induces thermogenic adipose tissue and reduces body fat. *Nature* **546**, 107-112 (2017). https://doi.org:10.1038/nature22342

31 Liu, L. *et al.* Histone methyltransferase MLL4 controls myofiber identity and muscle performance through MEF2 interaction. *J Clin Invest* **130**, 4710-4725 (2020). https://doi.org:10.1172/JCI136155

32 Ham, D. J. *et al.* Distinct and additive effects of calorie restriction and rapamycin in aging skeletal muscle. *Nat Commun* **13**, 2025 (2022).

https://doi.org:10.1038/s41467-022-29714-6

33 Greer, E. L. *et al.* An AMPK-FOXO pathway mediates longevity induced by a novel method of dietary restriction in C. elegans. *Current biology : CB* **17**, 1646-1656 (2007). https://doi.org:10.1016/j.cub.2007.08.047

34 Fang, E. F. *et al.* NAD(+) Replenishment Improves Lifespan and Healthspan in Ataxia Telangiectasia Models via Mitophagy and DNA Repair. *Cell metabolism* **24**, 566-581 (2016). https://doi.org:10.1016/j.cmet.2016.09.004

35 Bolger, A. M., Lohse, M. & Usadel, B. Trimmomatic: a flexible trimmer for Illumina sequence data. *Bioinformatics* **30**, 2114-2120 (2014). https://doi.org:10.1093/bioinformatics/btu170

36 Dobin, A. *et al.* STAR: ultrafast universal RNA-seq aligner. *Bioinformatics* **29**, 15-21 (2013). https://doi.org:10.1093/bioinformatics/bts635

37 Liao, Y., Smyth, G. K. & Shi, W. featureCounts: an efficient general purpose program for assigning sequence reads to genomic features. *Bioinformatics* **30**, 923-930 (2014). https://doi.org:10.1093/bioinformatics/btt656

38 Vacanti, N. M. *et al.* Regulation of substrate utilization by the mitochondrial pyruvate carrier. *Molecular cell* **56**, 425-435 (2014). https://doi.org:10.1016/j.molcel.2014.09.024

39 Millard, P., Letisse, F., Sokol, S. & Portais, J. C. IsoCor: correcting MS data in isotope labeling experiments. *Bioinformatics* **28**, 1294-1296 (2012). https://doi.org:10.1093/bioinformatics/bts127

40 Millard, P., Letisse, F., Sokol, S. & Portais, J. C. Correction of MS data for

naturally occurring isotopes in isotope labelling experiments. *Methods Mol Biol* **1191**, 197-207 (2014). https://doi.org:10.1007/978-1-4939-1170-7_12

41 Bajad, S. U. *et al.* Separation and quantitation of water soluble cellular metabolites by hydrophilic interaction chromatography-tandem mass spectrometry. *Journal of chromatography. A* **1125**, 76-88 (2006). https://doi.org:10.1016/j.chroma.2006.05.019

42 Preez, G. D. *et al.* Oxygen consumption rate of Caenorhabditis elegans as a high-throughput endpoint of toxicity testing using the Seahorse XF(e)96 Extracellular Flux Analyzer. *Scientific reports* **10**, 4239 (2020). https://doi.org:10.1038/s41598-020-61054-7

43 Amrhein, V., Greenland, S. & McShane, B. Scientists rise up against statistical significance. *Nature* **567**, 305-307 (2019). https://doi.org:10.1038/d41586-019-00857-9

44 Wasserstein, R. L., Schirm, A. L. & Lazar, N. A. Moving to a World Beyond "$p < 0.05$". *The American Statistician* **73**, 1-19 (2019). https://doi.org:10.1080/00031305.2019.1583913
