# Supplementary material for: AMPK-PDZD8-GLS1 axis mediates calorie restriction-induced lifespan extension
Source: Cell Res. 2024 Sep 19;34(11):806–9. doi: 10.1038/s41422-024-01021-3 (PMC11528062; doi:10.1038/s41422-024-01021-3)
Supplement: Supplementary file 2 — Supplementary information, Table S1 [file 41422_2024_1021_MOESM2_ESM.pdf]

Supplementary information, Table S1. Summary of lifespan analysis in worms[a,b]

Genotypes/ treatments	Mean life span (days)			Median life span (days)						P-value Vs saline control within each genotype (Mantel-CoX)
		95% confidence interval			95% confidence interval					
	Estimated life span ± s.e.m.	Lower bound	Upper bound	Estimated life span ± s.e.m.	Lower bound	Upper bound	N[c]	N[d]	N[e]	
**Fig. 1d**										
Pdzd-8$^{-/-}$ + PDZD8-WT	18.194 ± 0.353	17.503	18.886	18.000 ± 0.430	17.158	18.842	185	15	200	N/A
Pdzd-8$^{-/-}$ + PDZD8-WT + 2-DG	23.000 ± 0.477	22.064	23.935	24.000 ± 0.629	22.768	25.232	188	12	200	<0.001
Pdzd-8$^{-/-}$ + PDZD8-T527A	16.997 ± 0.399	16.215	17.779	18.000 ± 0.991	16.057	19.943	187	13	200	N/A
Pdzd-8$^{-/-}$ + PDZD8-T527A + 2-DG	15.568 ± 0.327	14.926	16.209	16.000 ± 0.490	15.039	16.961	184	16	200	<0.001
**Fig. 1e**										
CA-aak2;*Pdzd-8*$^{-/-}$ + PDZD8-T527A	19.293 ± 0.405	18.498	20.087	20.000 ± 0.585	18.853	21.147	180	20	200	N/A
CA-aak2;*Pdzd-8*$^{-/-}$ + PDZD8-WT	25.293 ± 0.512	24.289	26.296	28.000 ± 0.504	27.011	28.989	180	20	200	<0.001
**Fig. 1f**										
N2	19.946 ± 0.382	19.197	20.696	20.000 ± 0.532	18.957	21.043	193	7	200	N/A
N2 Ex PDZD8-T527E	24.592 ± 0.494	23.624	25.560	24.000 ± 0.708	22.612	25.388	193	7	200	<0.001
N2 Ex PDZD8-T527D	20.044 ± 0.389	19.280	20.807	20.000 ± 0.480	19.059	20.941	193	7	200	0.774
**Fig. 1g (left)**										
Pdzd-8$^{-/-}$ + PDZD8-WT	19.603 ± 0.375	18.868	20.337	20.000 ± 0.448	19.123	20.877	194	6	200	N/A
Pdzd-8$^{-/-}$ + PDZD8-WT + 2-DG	24.790 ± 0.483	23.842	25.737	25.000 ± 0.722	22.585	25.415	189	11	200	<0.001
Pdzd-8$^{-/-}$ + PDZD8-T527D	20.083 ± 0.380	19.337	20.828	20.000 ± 0.462	19.094	20.906	193	7	200	N/A
Pdzd-8$^{-/-}$ + PDZD8-T527D + 2-DG	19.852 ± 0.395	19.078	20.626	20.000 ± 0.544	18.933	21.067	192	8	200	0.679
**Fig. 1g (right)**										
Pdzd-8$^{-/-}$ + PDZD8-WT	19.678 ± 0.380	18.934	20.422	20.000 ± 0.431	19.156	20.844	190	10	200	N/A
Pdzd-8$^{-/-}$ + PDZD8-WT + 2-DG	24.399 ± 0.486	23.447	25.352	24.000 ± 0.490	23.039	24.961	191	9	200	<0.001
Pdzd-8$^{-/-}$ + PDZD8-T527E	24.123 ± 0.498	23.147	25.100	24.000 ± 0.762	22.507	25.493	191	9	200	N/A
Pdzd-8$^{-/-}$ + PDZD8-T527E + 2-DG	24.256 ± 0.489	23.297	25.215	24.000 ± 0.471	23.076	24.924	190	10	200	0.920
**Fig. 1h**										
Pdzd-8$^{-/-}$ + PDZD8-WT	18.763 ± 0.394	17.991	19.536	20.000 ± 0.735	18.559	21.411	186	14	200	N/A
Pdzd-8$^{-/-}$ + PDZD8-WT + CR	25.067 ± 0.526	24.036	26.099	26.000 ± 0.548	24.925	27.075	184	16	200	<0.001
Pdzd-8$^{-/-}$ + PDZD8-T527A	19.114 ± 0.391	18.348	19.880	20.000 ± 0.559	18.904	21.096	179	21	200	N/A
Pdzd-8$^{-/-}$ + PDZD8-T527A + CR	16.428 ± 0.277	15.885	16.971	18.000 ± 0.362	17.290	18.710	184	16	200	<0.001
**Fig. 1j**										
glna-null + KGA-WT	18.816 ± 0.405	18.023	19.609	18.000 ± 0.684	16.659	19.341	177	23	200	N/A
glna-null + KGA-WT + 2-DG	21.750 ± 0.434	20.899	22.600	22.000 ± 0.454	21.111	22.889	188	20	200	<0.001
glna-null + KGA-33A	18.330 ± 0.395	17.556	19.104	18.000 ± 0.776	16.480	19.520	187	13	200	N/A
glna-null + KGA-33A + 2-DG	18.502 ± 0.395	17.728	19.276	18.000 ± 0.795	16.442	19.558	186	14	200	0.487
**Fig. 1i**										
N2	18.030 ± 0.376	17.294	18.766	18.000 ± 0.482	17.056	18.944	184	16	200	N/A
N2 + 2-DG	21.282 ± 0.469	20.363	22.202	22.000 ± 0.605	20.185	23.185	187	13	200	<0.001
glna-null	17.710 ± 0.353	17.018	18.402	18.000 ± 0.447	17.124	18.876	189	11	200	N/A
glna-null + 2DG	18.084 ± 0.373	17.354	18.815	18.000 ± 0.482	17.056	18.944	188	12	200	0.920
**Fig. 1k**										
N2	18.215 ± 0.356	17.517	18.912	18.000 ± 0.440	17.137	18.863	187	13	200	N/A
N2 + CR	21.786 ± 0.437	20.931	22.642	22.000 ± 0.625	20.774	23.226	186	14	200	<0.001
glna-null	18.229 ± 0.365	17.514	18.943	18.000 ± 0.448	17.121	18.879	185	15	200	N/A
glna-null + CR	18.415 ± 0.377	17.676	19.154	18.000 ± 0.514	16.993	19.007	181	19	200	0.895
**Fig. 1l**										
glna-null + KGA-WT	19.414 ± 0.406	18.619	20.210	20.000 ± 0.469	19.081	20.919	179	21	200	N/A
glna-null + KGA-WT + CR	25.293 ± 0.5212	24.289	26.296	26.000 ± 0.462	27.011	26.905	178	22	200	<0.001
glna-null + KGA-33A	18.971 ± 0.409	18.169	19.772	20.000 ± 0.525	18.971	21.029	182	18	200	N/A
glna-null + KGA-33A + CR	19.140 ± 0.380	18.396	19.885	20.000 ± 0.521	18.978	21.022	179	21	200	0.404
**Fig. 1q (left)**										
N2 + CR	23.900 ± 0.537	22.847	24.953	24.000 ± 0.562	22.898	25.102	200	0	200	N/A
N2 + CR + NAC	19.866 ± 0.376	19.129	20.602	20.000 ± 0.347	19.319	20.681	193	7	200	<0.001

Supplementary information, Table S1. Summary of lifespan analysis in worms (cont.)[a,b]

	Mean life span (days)			Median life span (days)						P-value Vs saline control within each genotype (Mantel-CoX)
		95% confidence interval			95% confidence interval					
Genotypes/ treatments	Estimated life span ± s.e.m.	Lower bound	Upper bound	Estimated life span ± s.e.m.	Lower bound	Upper bound	N[c]	N[d]	N[e]	
				Fig. 1q (right)						
N2	21.757 ± 0.372	21.029	22.485	22.000 ± 0.311	21.390	22.610	190	10	200	N/A
N2 + NAC	21.658 ± 0.402	20.869	22.446	22.000 ± 0.437	21.143	22.857	189	11	200	N/A
				Supplementary information, Fig. S2a						
N2	20.554 ± 0.374	19.820	21.288	20.000 ± 0.434	19.149	20.851	185	15	200	N/A
N2 + 2-DG	24.984 ± 0.465	24.074	25.895	24.000 ± 0.651	22.724	25.276	188	12	200	<0.001
Pdzd-8^{-}	20.340 ± 0.368	19.619	21.061	20.000 ± 0.457	19.104	20.896	186	14	200	N/A
Pdzd-8$^{-/-}$ + 2-DG	20.318 ± 0.378	19.578	21.058	20.000 ± 0.494	19.031	20.969	180	20	200	0.894
				Supplementary information, Fig. S2d						
N2	19.835 ± 0.367	19.115	20.554	20.000 ± 0.427	19.162	20.838	193	7	200	N/A
N2 + CR	26.010 ± 0.524	24.983	27.037	26.000 ± 0.748	24.533	27.467	194	6	200	<0.001
Pdzd-8$^{-/-}$	19.653 ± 0.367	18.935	20.372	20.000 ± 0.475	19.068	20.932	196	4	200	N/A
Pdzd-8$^{-/-}$ + CR	19.803 ± 0.377	19.065	20.541	20.000 ± 0.569	18.885	21.115	193	7	200	0.720

[a]Independent repeats of each lifespan experiment were performed. Data from representative experiments are shown.
[b]Lifespan data sets within each panel of this table were done in parallel and statistical analyses was done within the data set.
[c]Number of worms scored (death events).
[d]Number of worms censored.
[e]Total number of worms.

Supplementary information, Table S1. Summary of healthspan analysis in worms[a,b]

Genotypes/ treatments	Mean life span (hours)			Median life span (hours)						P-value Vs saline control within each genotype (Mantel-CoX)
	Estimated life span ± s.e.m.	95% confidence interval		Estimated life span ± s.e.m.	95% confidence interval					
		Lower bound	Upper bound		Lower bound	Upper bound	N[c]	N[d]	N[e]	
**Fig. 1o**										
Pdzd-8$^{-/-}$ + PDZD8-WT	10.231 ± 0.558	9.138	11.324	11.000 ± 0.929	9.179	12.821	51	9	60	N/A
Pdzd-8$^{-/-}$ + PDZD8-WT + CR	14.878 ± 0.663	13.578	16.177	16.000 ± 0.779	14.474	17.526	47	13	60	<0.001
Pdzd-8$^{-/-}$ + PDZD8-T527A	10.268 ± 0.493	9.302	11.234	11.000 ± 0.782	9.467	12.533	54	6	60	N/A
Pdzd-8$^{-/-}$ + PDZD8-T527A + CR	10.947 ± 0.487	9.992	11.901	12.000 ± 0.551	10.920	13.080	47	13	60	0.829
**Fig. 1p**										
glna-null + KGA-WT	10.485 ± 0.513	9.480	11.490	11.000 ± 0.924	9.189	12.811	47	13	60	N/A
glna-null + KGA-WT + CR	14.233 ± 0.722	12.818	15.647	15.000 ± 0.867	13.301	16.699	49	11	60	<0.001
glna-null + KGA-33A	10.804 ± 0.429	9.964	11.644	11.000 ± 0.575	9.874	12.126	48	12	60	N/A
glna-null + KGA-33A + CR	10.631 ± 0.491	9.668	11.595	11.000 ± 0.564	9.894	12.106	48	12	60	0.969
**Supplementary information, Fig. S2g**										
Pdzd-8$^{-/-}$ + PDZD8-WT	9.584 ± 0.561	8.485	10.683	10.000 ± 0.859	8.316	11.684	49	11	60	N/A
Pdzd-8$^{-/-}$ + PDZD8-WT + 2-DG	13.767 ± 0.762	12.274	15.260	15.000 ± 0.758	13.514	16.486	50	10	60	<0.001
Pdzd-8$^{-/-}$ + PDZD8-T527A	10.565 ± 0.464	9.654	11.475	11.000 ± 0.627	9.771	12.229	49	11	60	N/A
Pdzd-8$^{-/-}$ + PDZD8-T527A + 2-DG	10.407 ± 0.523	9.381	11.432	11.000 ± 0.732	9.565	12.435	48	12	60	0.430
**Supplementary information, Fig. S2h**										
glna-null + KGA-WT	10.532 ± 0.510	9.533	11.531	11.000 ± 0.972	9.095	12.905	48	12	60	N/A
glna-null + KGA-WT + 2-DG	10.867 ± 0.460	13.356	16.067	16.000 ± 0.842	20.774	17.651	47	13	60	<0.001
glna-null + KGA-33A	11.494 ± 0.453	10.605	12.383	12.000 ± 0.551	10.919	13.081	45	15	60	N/A
glna-null + KGA-33A + 2-DG	10.867 ± 0.460	9.965	11.770	11.000 ± 0.518	9.984	12.016	45	15	60	0.024

[a]Independent repeats of each health span experiment were performed. Data from representative experiments are shown.
[b]Health span data sets within each panel of this table were done in parallel and statistical analyses was done within the data set.
[c]Number of worms scored (death events).
[d]Number of worms censored.
[e]Total number of worms.
