# Supplementary material for: AMPK-PDZD8-GLS1 axis mediates calorie restriction-induced lifespan extension
Source: Cell Res. 2024 Sep 19;34(11):806–9. doi: 10.1038/s41422-024-01021-3 (PMC11528062; doi:10.1038/s41422-024-01021-3)

Full scans.

Blots, as shown in this figure, were cut into slices before incubation with primary antibodies. The Pierce™ Prestained Protein MW Marker, Cat. 26612, from ThermoFisher Scientific, was used as the protein marker.

Fig. 1a (left)

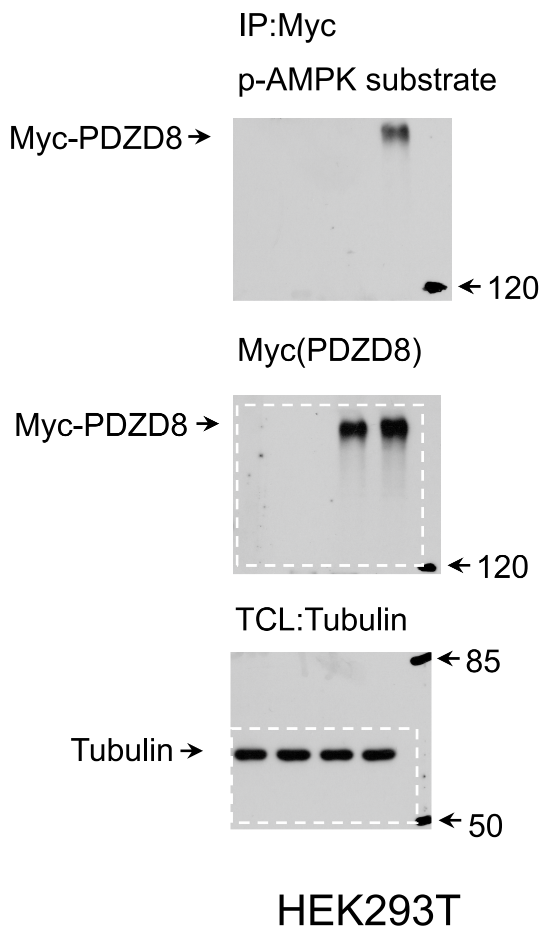

Fig. 1a (right)

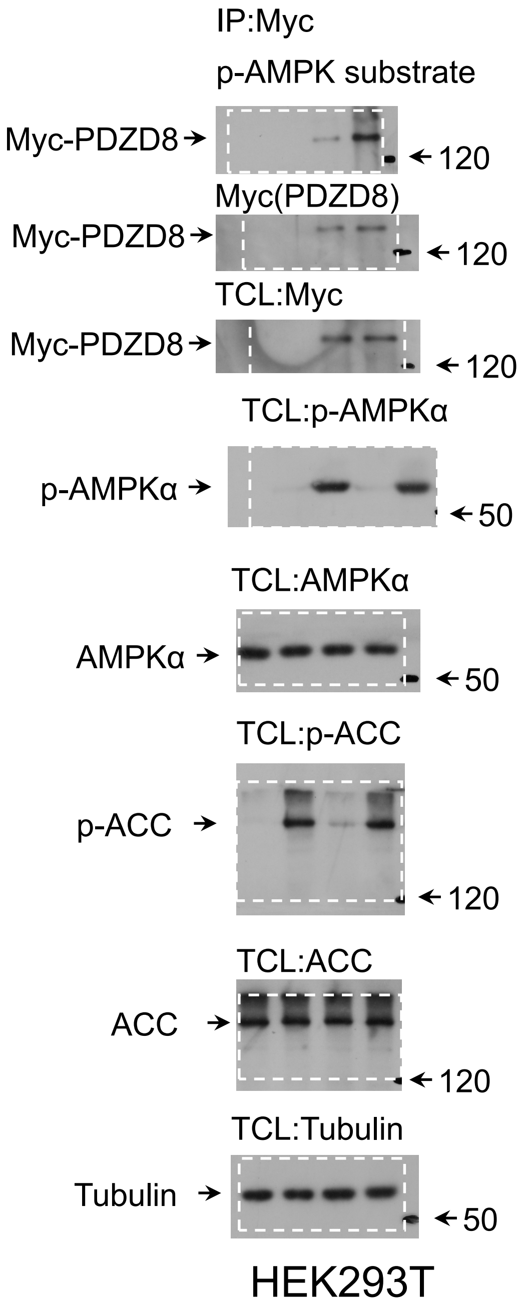

Fig. S1b

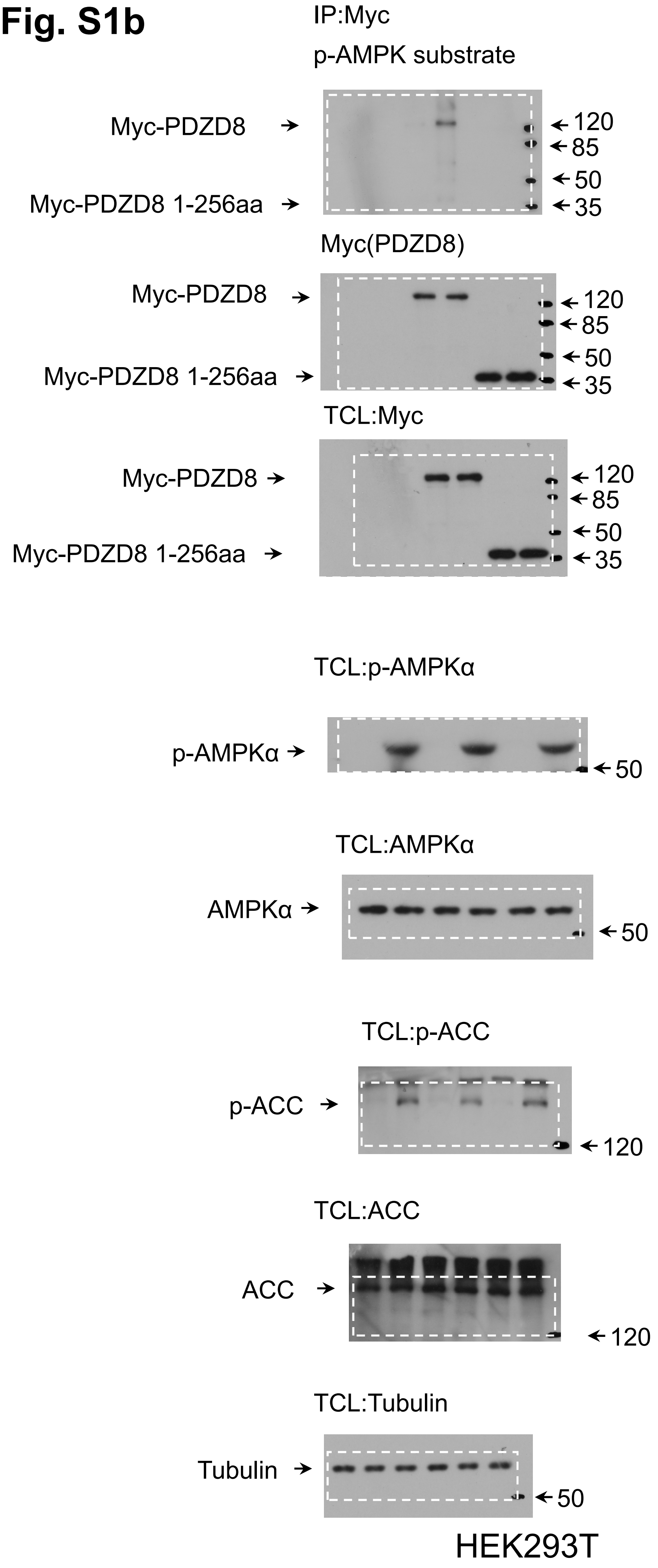
IP:Myc
p-AMPK substrate
Myc-PDZD8
Myc-PDZD8 1-256aa
120
85
50
35
Myc(PDZD8)
Myc-PDZD8
Myc-PDZD8 1-256aa
120
85
50
35
TCL:Myc
Myc-PDZD8
Myc-PDZD8 1-256aa
120
85
50
35
TCL:p-AMPKα
p-AMPKα
50
TCL:AMPKα
AMPKα
50
TCL:p-ACC
p-ACC
120
TCL:ACC
ACC
120
TCL:Tubulin
Tubulin
50
HEK293T

Fig. S1b

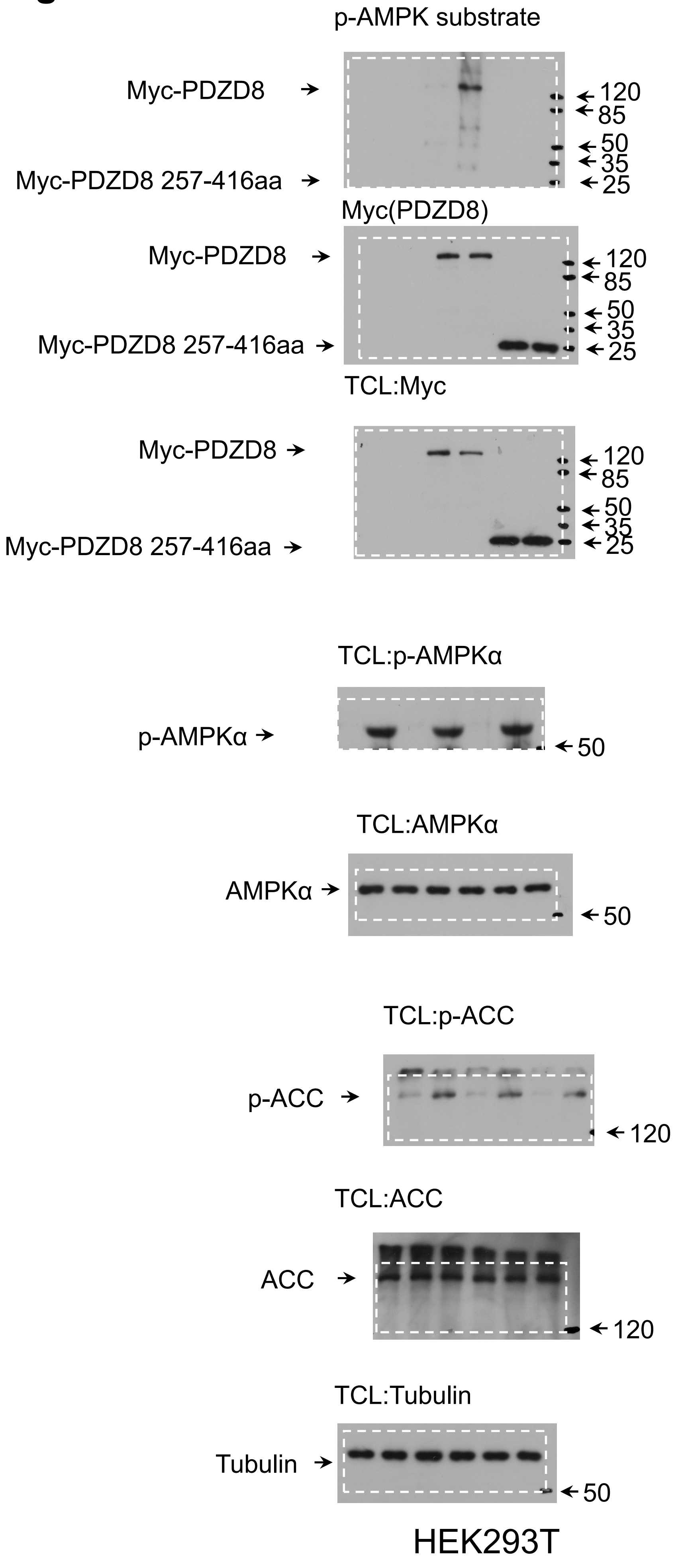
IP:Myc
p-AMPK substrate
Myc-PDZD8
Myc-PDZD8 257-416aa
120
85
50
35
25
Myc(PDZD8)
Myc-PDZD8
Myc-PDZD8 257-416aa
120
85
50
35
25
TCL:Myc
Myc-PDZD8
Myc-PDZD8 257-416aa
120
85
50
35
25
TCL:p-AMPKα
p-AMPKα
50
TCL:AMPKα
AMPKα
50
TCL:p-ACC
p-ACC
120
TCL:ACC
ACC
120
TCL:Tubulin
Tubulin
50
HEK293T

Fig. S1b

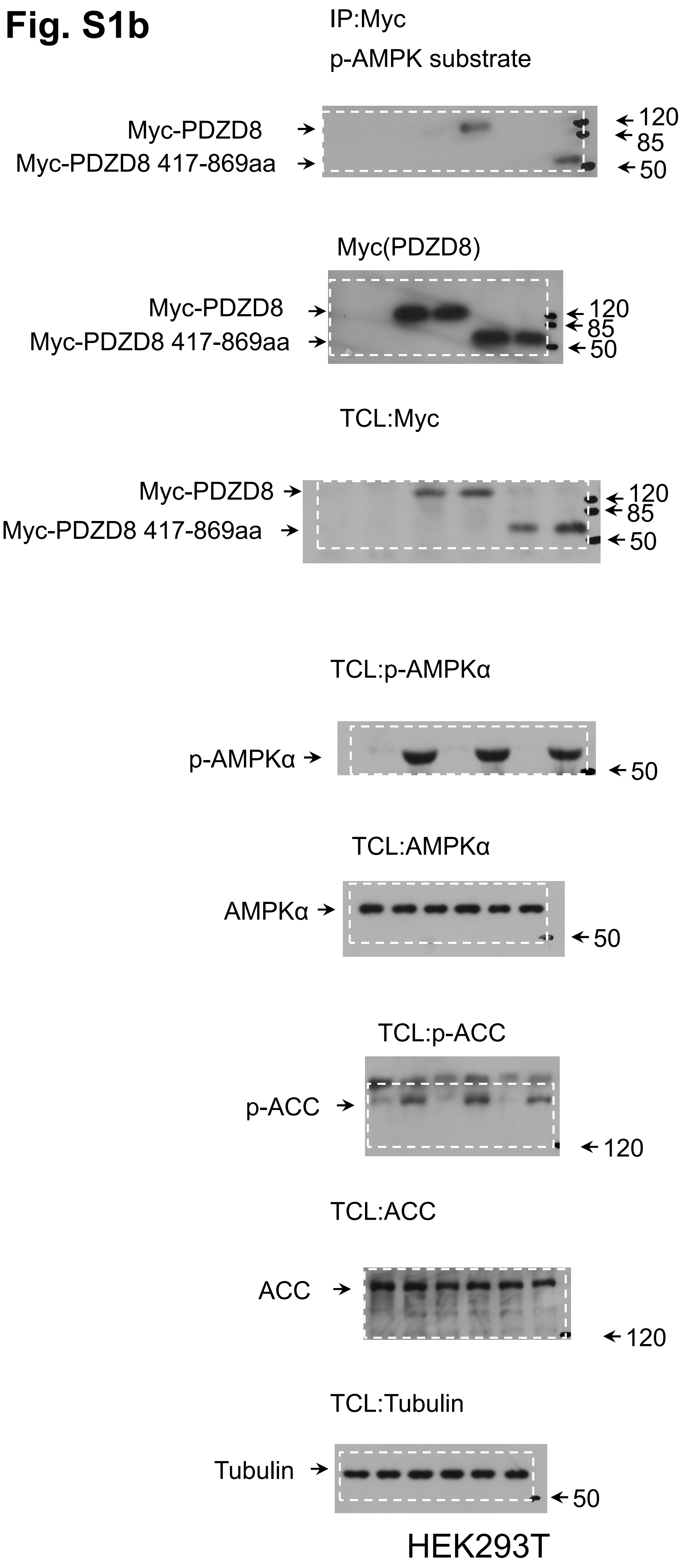
IP:Myc
p-AMPK substrate
Myc-PDZD8
Myc-PDZD8 417-869aa
120
85
50
Myc(PDZD8)
Myc-PDZD8
Myc-PDZD8 417-869aa
120
85
50
TCL:Myc
Myc-PDZD8
Myc-PDZD8 417-869aa
120
85
50
TCL:p-AMPKα
p-AMPKα
50
TCL:AMPKα
AMPKα
50
TCL:p-ACC
p-ACC
120
TCL:ACC
ACC
120
TCL:Tubulin
Tubulin
50
HEK293T

Fig. S1b

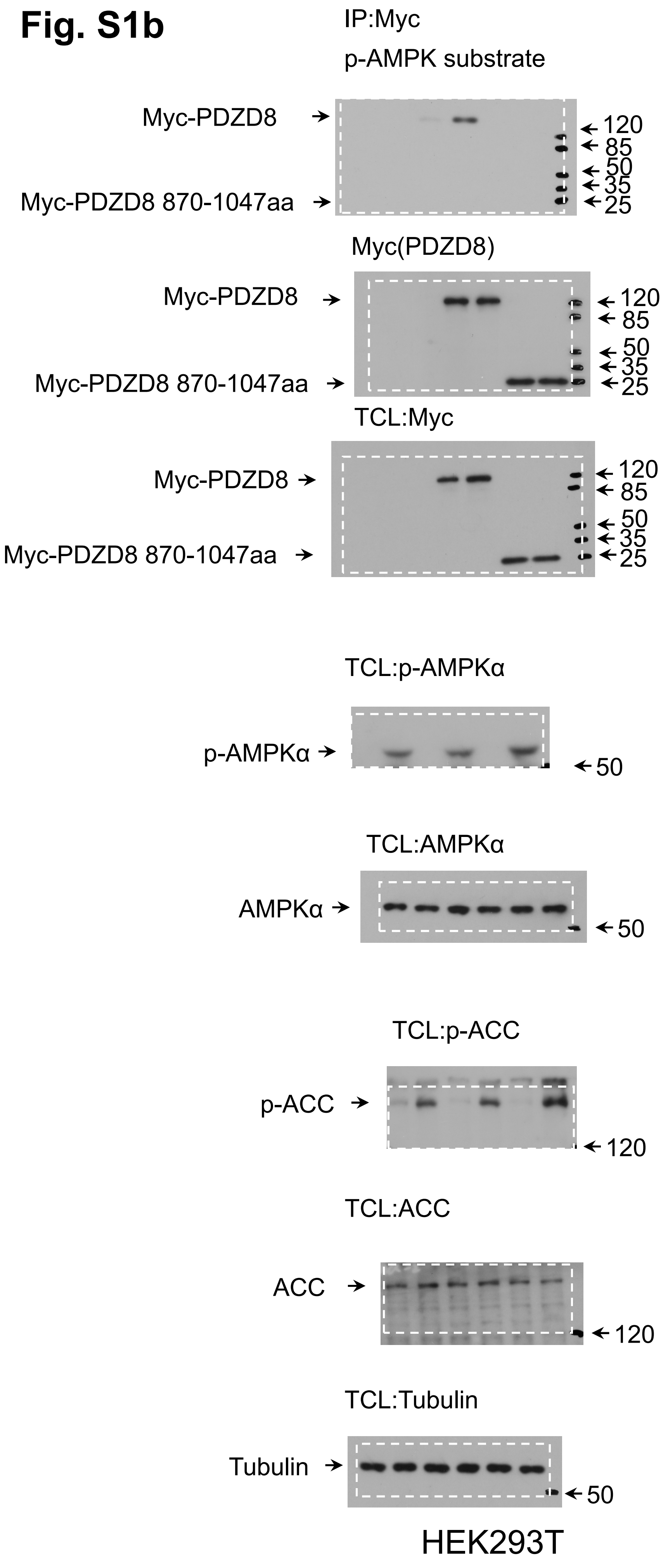
IP:Myc
p-AMPK substrate
Myc-PDZD8
Myc-PDZD8 870-1047aa
120
85
50
35
25
Myc(PDZD8)
Myc-PDZD8
Myc-PDZD8 870-1047aa
120
85
50
35
25
TCL:Myc
Myc-PDZD8
Myc-PDZD8 870-1047aa
120
85
50
35
25
TCL:p-AMPKα
p-AMPKα
50
TCL:AMPKα
AMPKα
50
TCL:p-ACC
p-ACC
120
TCL:ACC
ACC
120
TCL:Tubulin
Tubulin
50
HEK293T

Fig. S1b

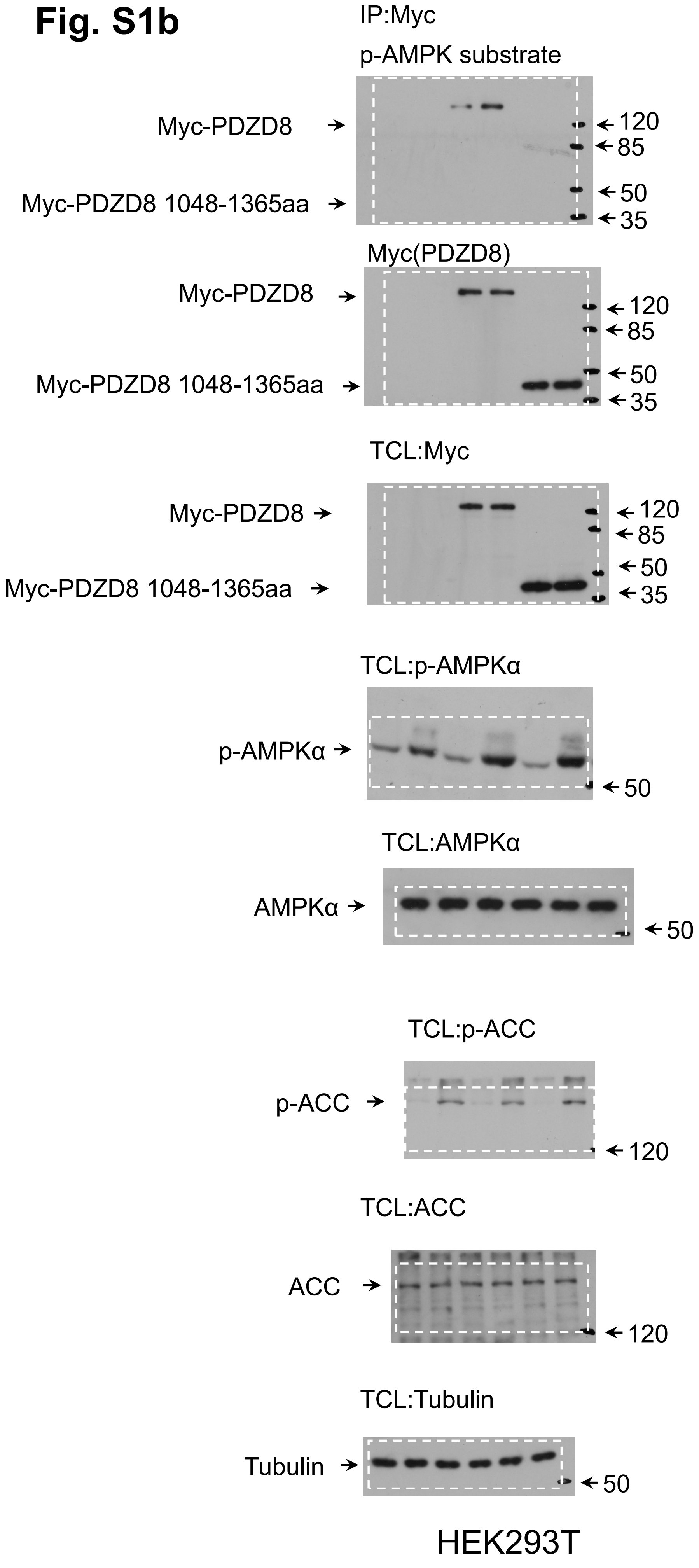
IP:Myc
p-AMPK substrate
Myc-PDZD8
Myc-PDZD8 1048-1365aa
120
85
50
35
Myc(PDZD8)
Myc-PDZD8
Myc-PDZD8 1048-1365aa
120
85
50
35
TCL:Myc
Myc-PDZD8
Myc-PDZD8 1048-1365aa
120
85
50
35
TCL:p-AMPKα
p-AMPKα
50
TCL:AMPKα
AMPKα
50
TCL:p-ACC
p-ACC
120
TCL:ACC
ACC
120
TCL:Tubulin
Tubulin
50
HEK293T

Fig. S1b

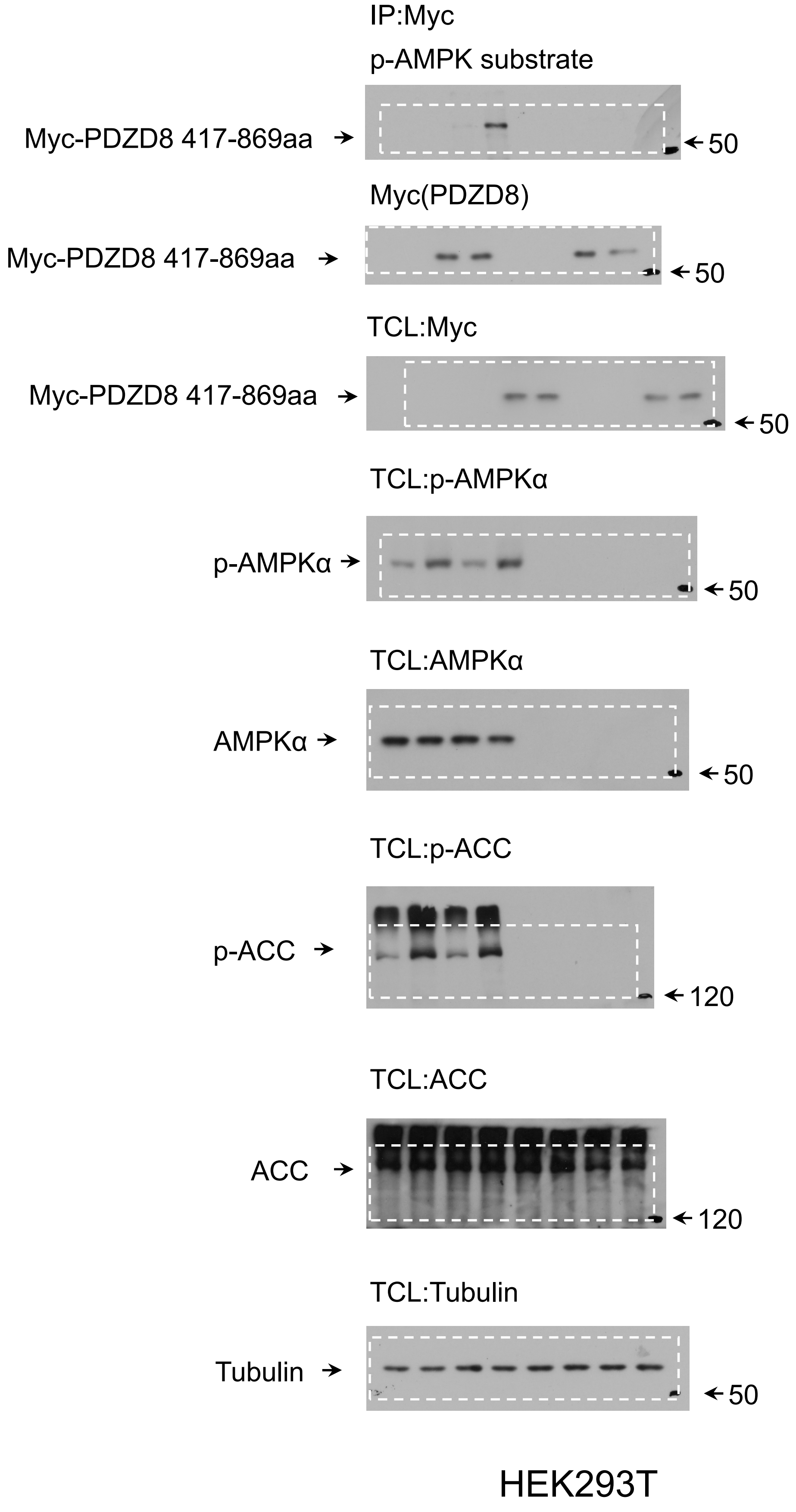
IP:Myc
p-AMPK substrate
Myc-PDZD8 417-869aa
50
Myc(PDZD8)
Myc-PDZD8 417-869aa
50
TCL:Myc
Myc-PDZD8 417-869aa
50
TCL:p-AMPKα
p-AMPKα
50
TCL:AMPKα
AMPKα
50
TCL:p-ACC
p-ACC
120
TCL:ACC
ACC
120
TCL:Tubulin
Tubulin
50
HEK293T

Fig. S1c

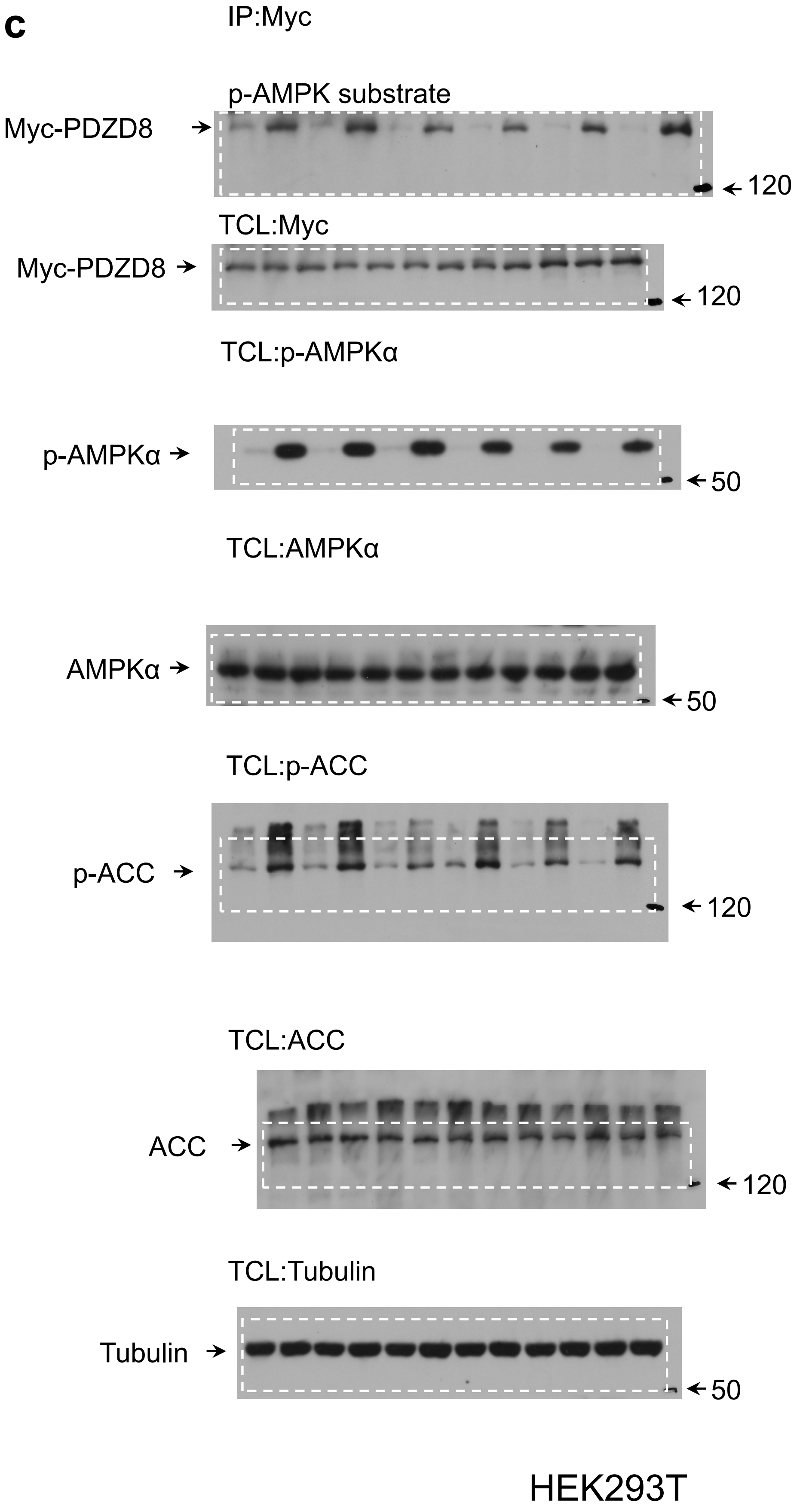
IP:Myc
p-AMPK substrate
Myc-PDZD8
120
TCL:Myc
Myc-PDZD8
120
TCL:p-AMPKα
p-AMPKα
50
TCL:AMPKα
AMPKα
50
TCL:p-ACC
p-ACC
120
TCL:ACC
ACC
120
TCL:Tubulin
Tubulin
50
HEK293T

Fig. S1c

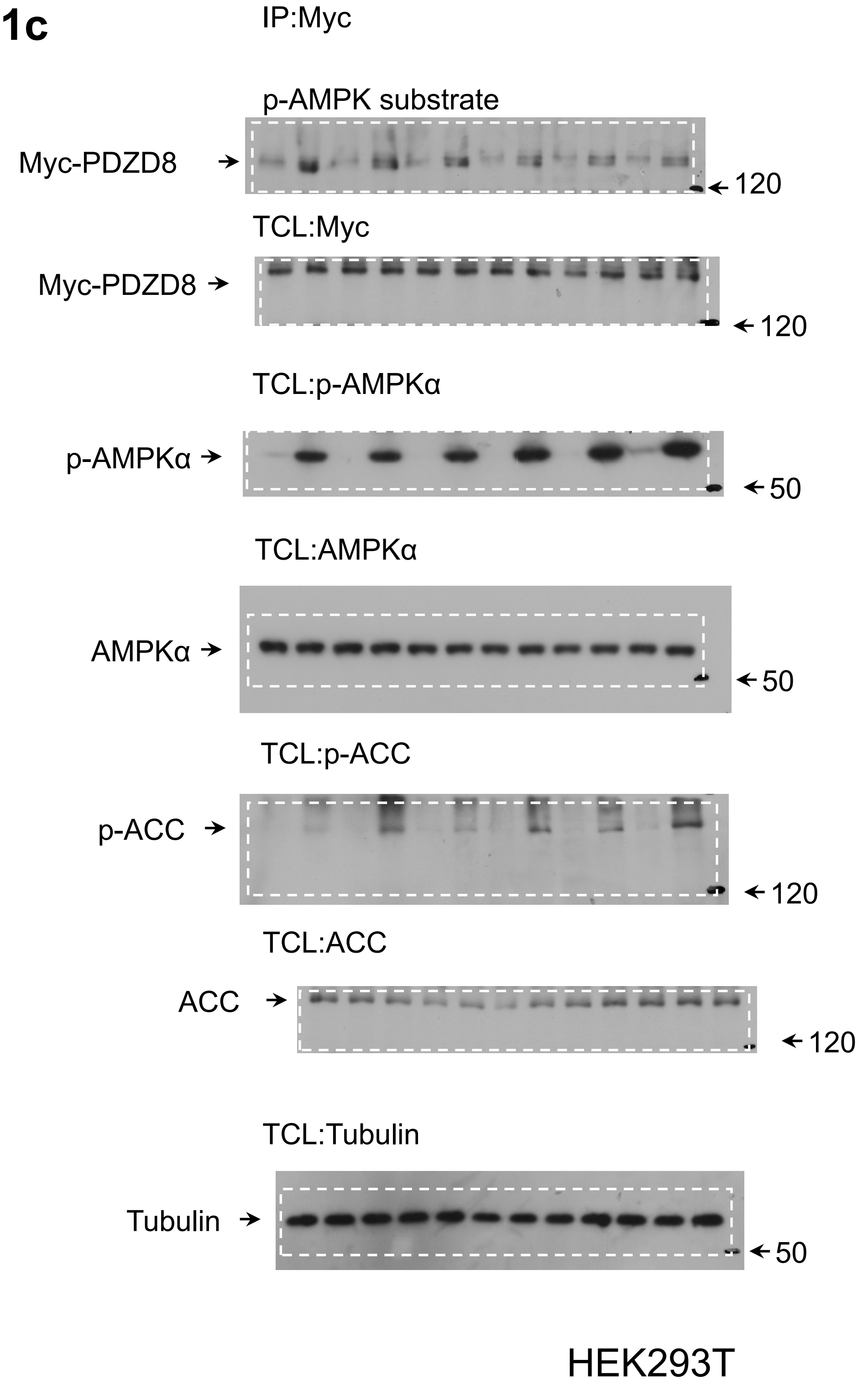
IP:Myc
p-AMPK substrate
Myc-PDZD8
120
TCL:Myc
Myc-PDZD8
120
TCL:p-AMPKα
p-AMPKα
50
TCL:AMPKα
AMPKα
50
TCL:p-ACC
p-ACC
120
TCL:ACC
ACC
120
TCL:Tubulin
Tubulin
50
HEK293T

Fig. S1c

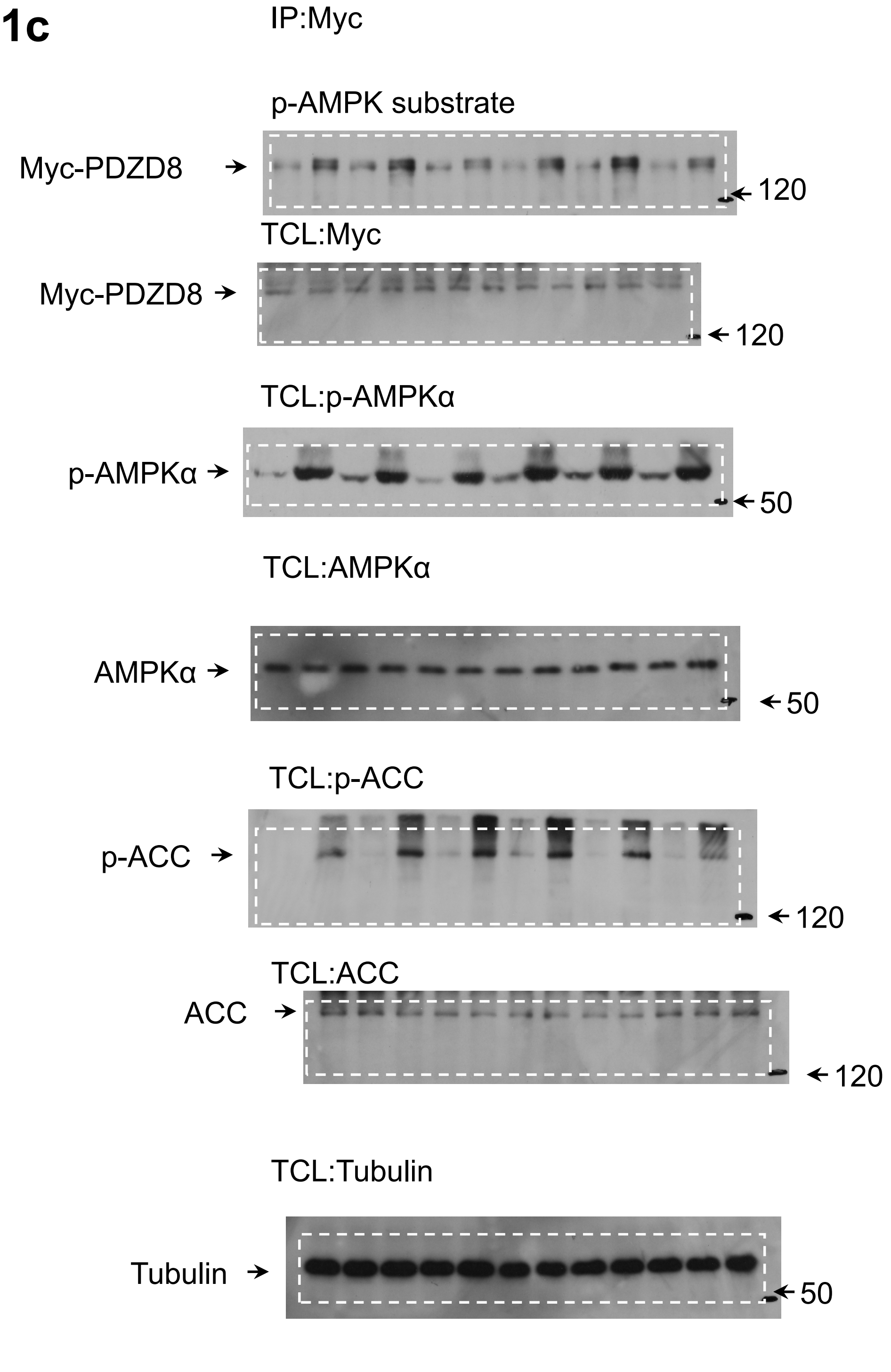
IP:Myc
p-AMPK substrate
Myc-PDZD8
120
TCL:Myc
Myc-PDZD8
120
TCL:p-AMPKα
p-AMPKα
50
TCL:AMPKα
AMPKα
50
TCL:p-ACC
p-ACC
120
TCL:ACC
ACC
120
TCL:Tubulin
Tubulin
50
HEK293T

Fig. S1c

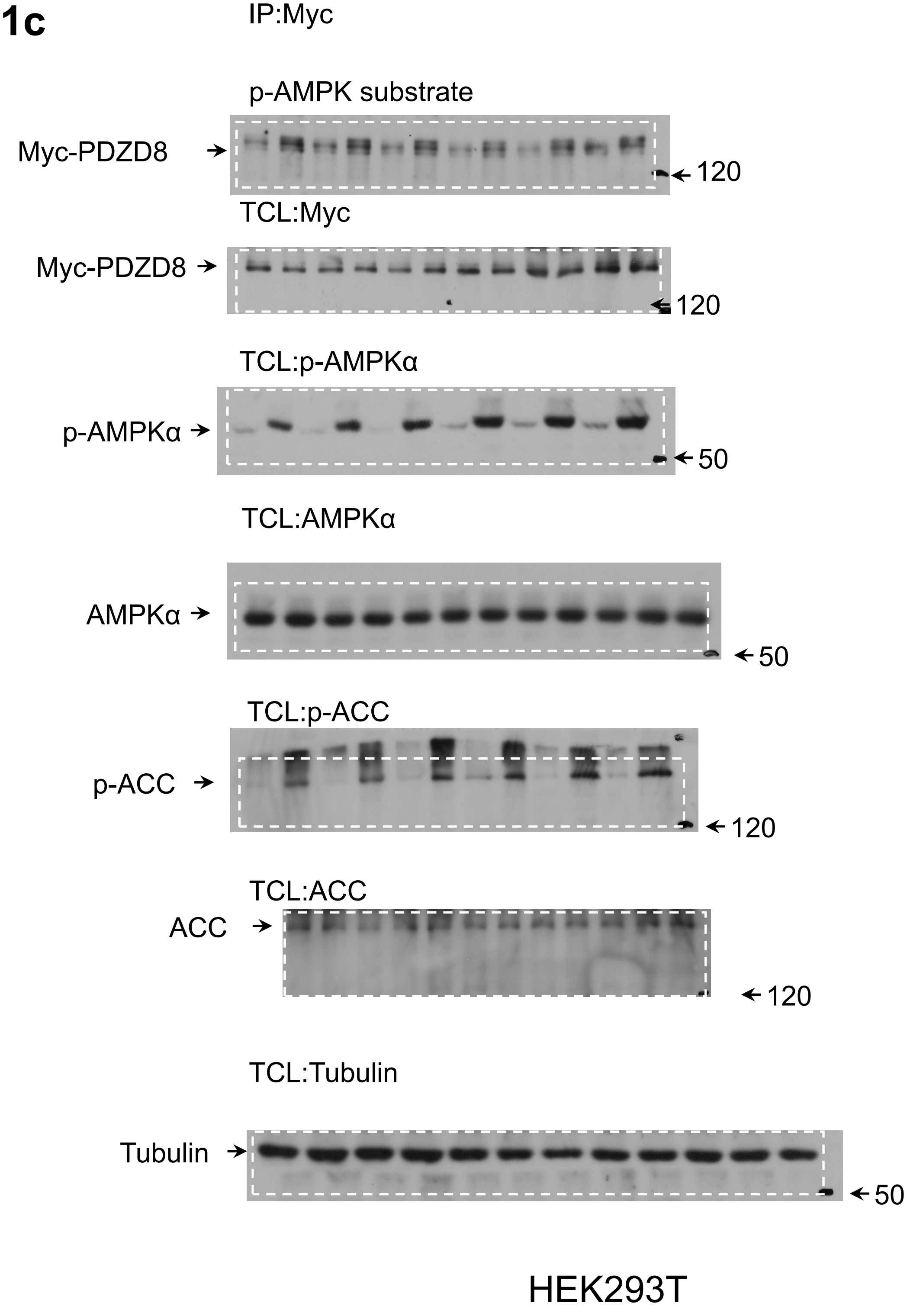
IP:Myc
p-AMPK substrate
Myc-PDZD8
120
TCL:Myc
Myc-PDZD8
120
TCL:p-AMPKα
p-AMPKα
50
TCL:AMPKα
AMPKα
50
TCL:p-ACC
p-ACC
120
TCL:ACC
ACC
120
TCL:Tubulin
Tubulin
50
HEK293T

Fig. S1c

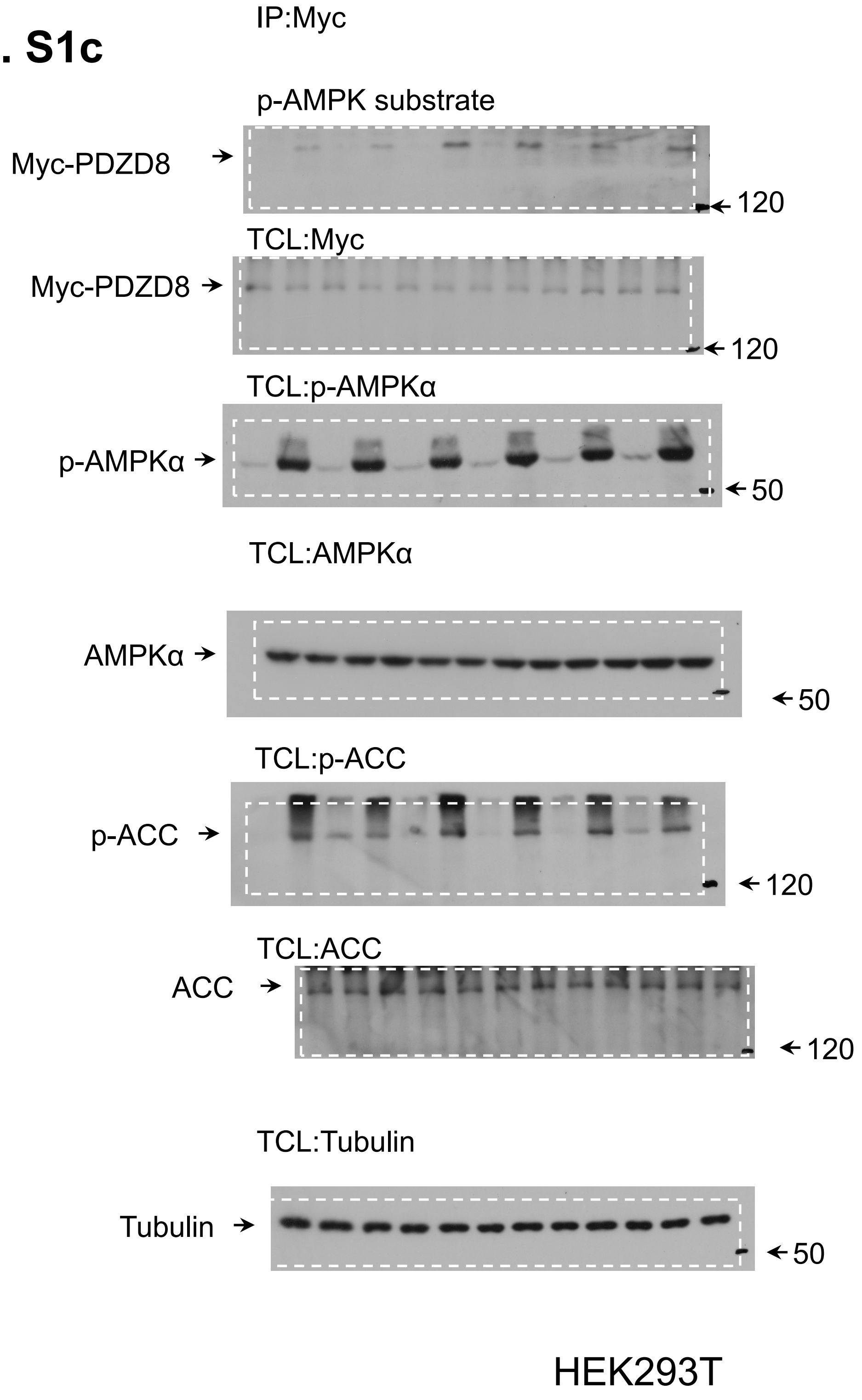
IP:Myc
p-AMPK substrate
Myc-PDZD8
120
TCL:Myc
Myc-PDZD8
120
TCL:p-AMPKα
p-AMPKα
50
TCL:AMPKα
AMPKα
50
TCL:p-ACC
p-ACC
120
TCL:ACC
ACC
120
TCL:Tubulin
Tubulin
50
HEK293T

Fig. S1c

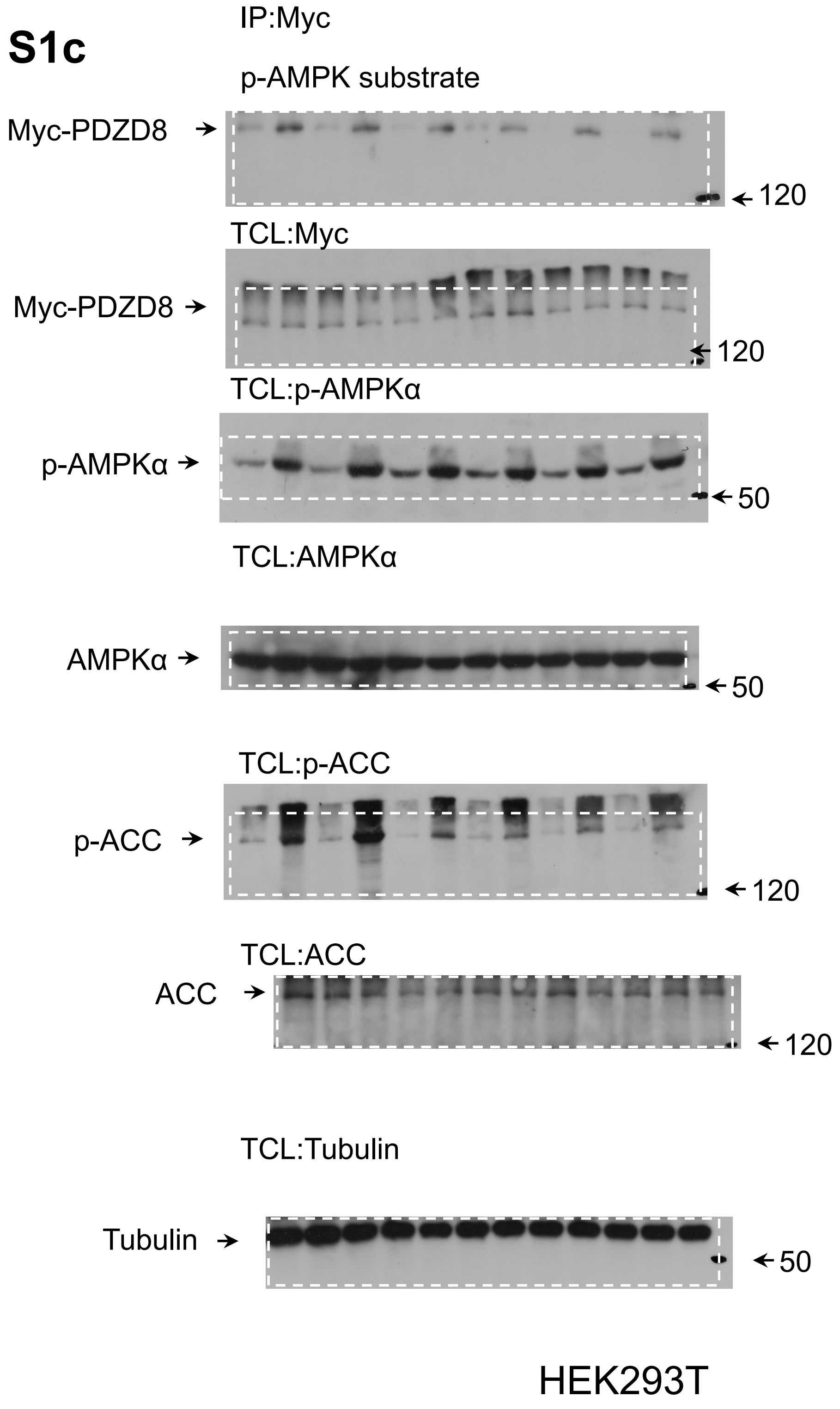
IP:Myc
p-AMPK substrate
Myc-PDZD8
120
TCL:Myc
Myc-PDZD8
120
TCL:p-AMPKα
p-AMPKα
50
TCL:AMPKα
AMPKα
50
TCL:p-ACC
p-ACC
120
TCL:ACC
ACC
120
TCL:Tubulin
Tubulin
50
HEK293T

Fig. S1c

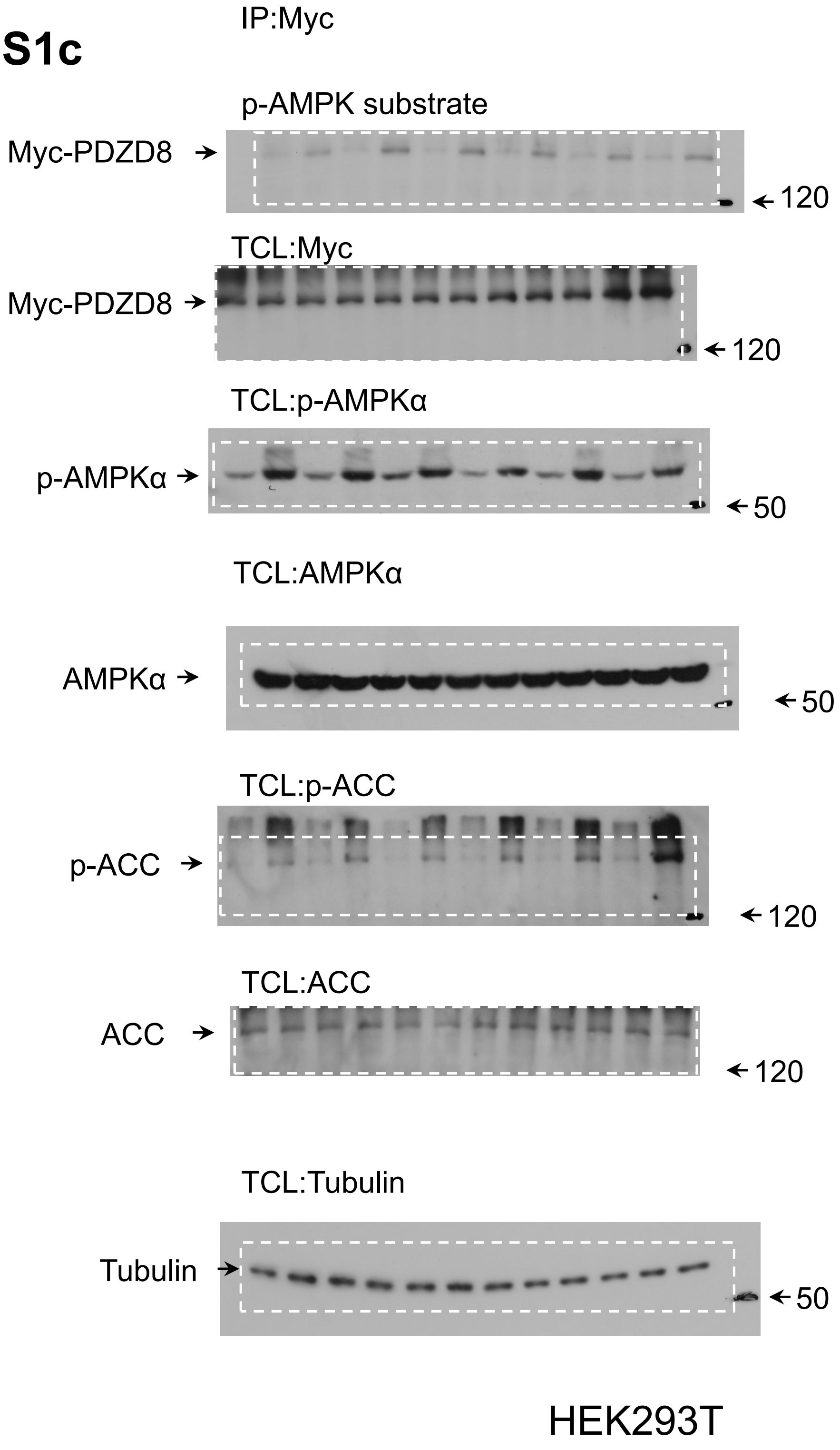
IP:Myc
p-AMPK substrate
Myc-PDZD8
120
TCL:Myc
Myc-PDZD8
120
TCL:p-AMPKα
p-AMPKα
50
TCL:AMPKα
AMPKα
50
TCL:p-ACC
p-ACC
120
TCL:ACC
ACC
120
TCL:Tubulin
Tubulin
50
HEK293T

Fig. S1c

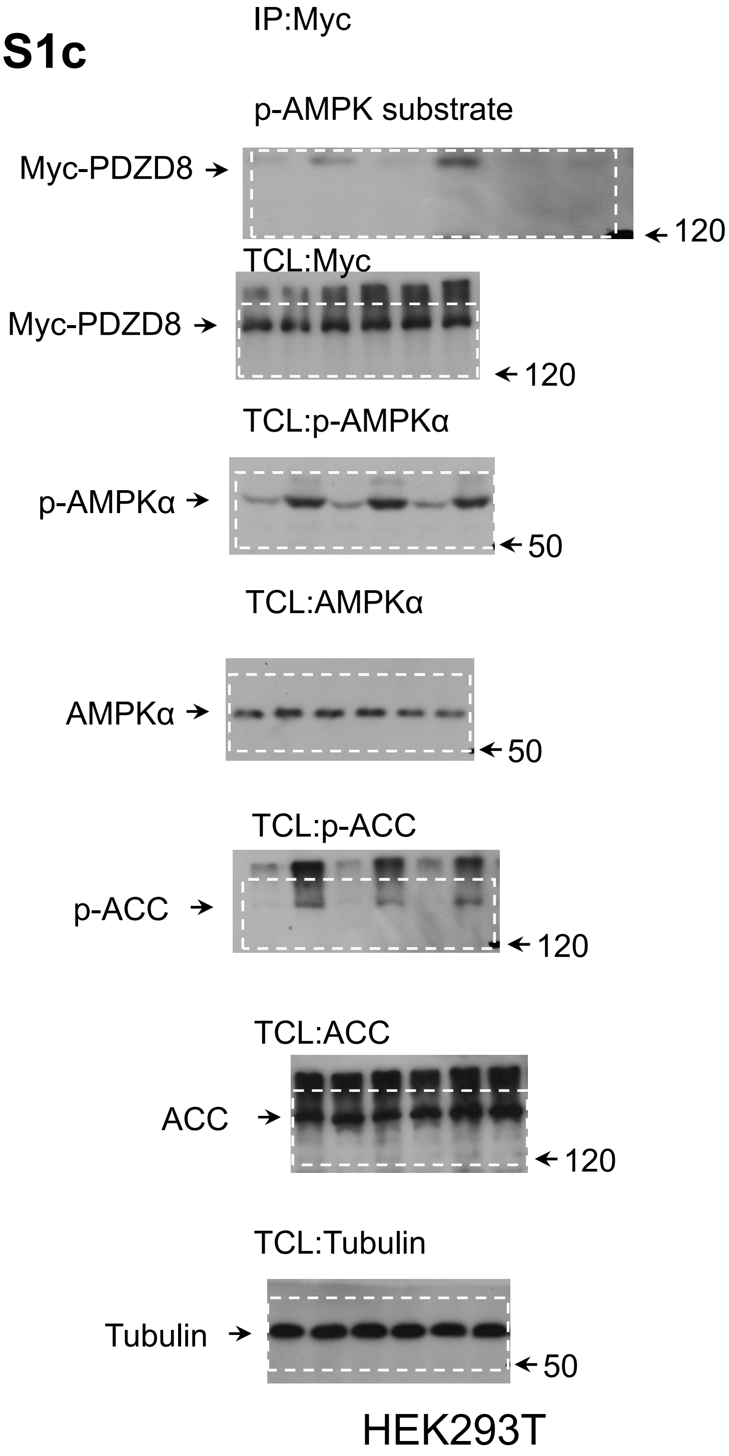
IP:Myc
p-AMPK substrate
Myc-PDZD8
120
TCL:Myc
Myc-PDZD8
120
TCL:p-AMPKα
p-AMPKα
50
TCL:AMPKα
AMPKα
50
TCL:p-ACC
p-ACC
120
TCL:ACC
ACC
120
TCL:Tubulin
Tubulin
50
HEK293T

Fig. S1c

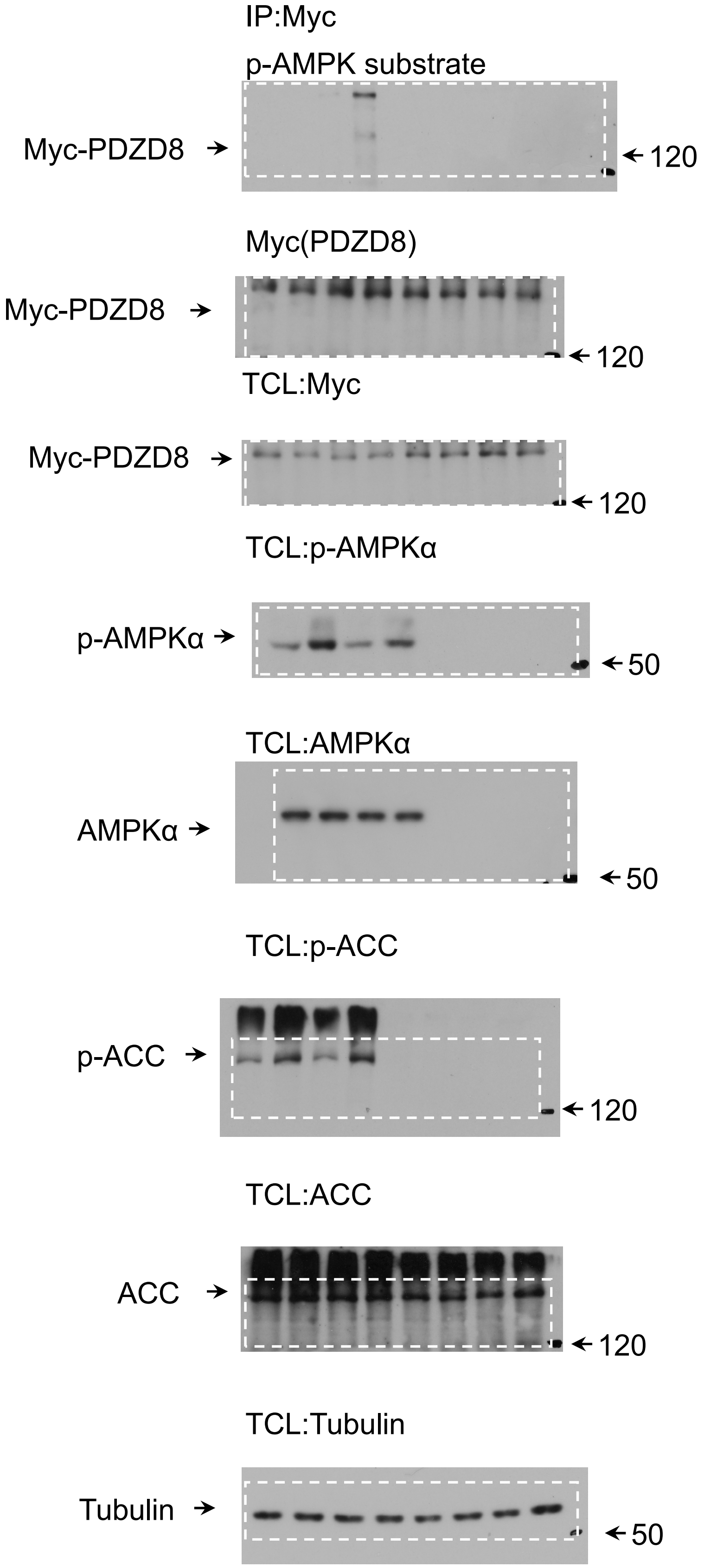

HEK293T

Fig. S1d

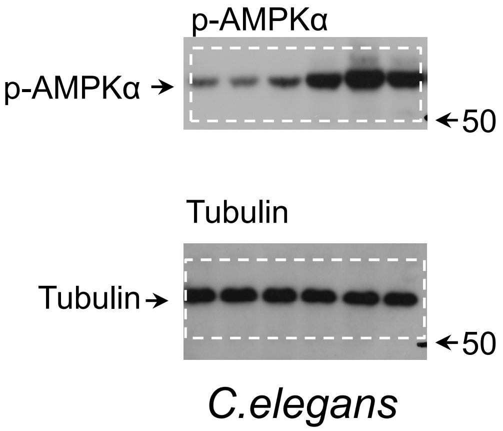

Fig. S1g

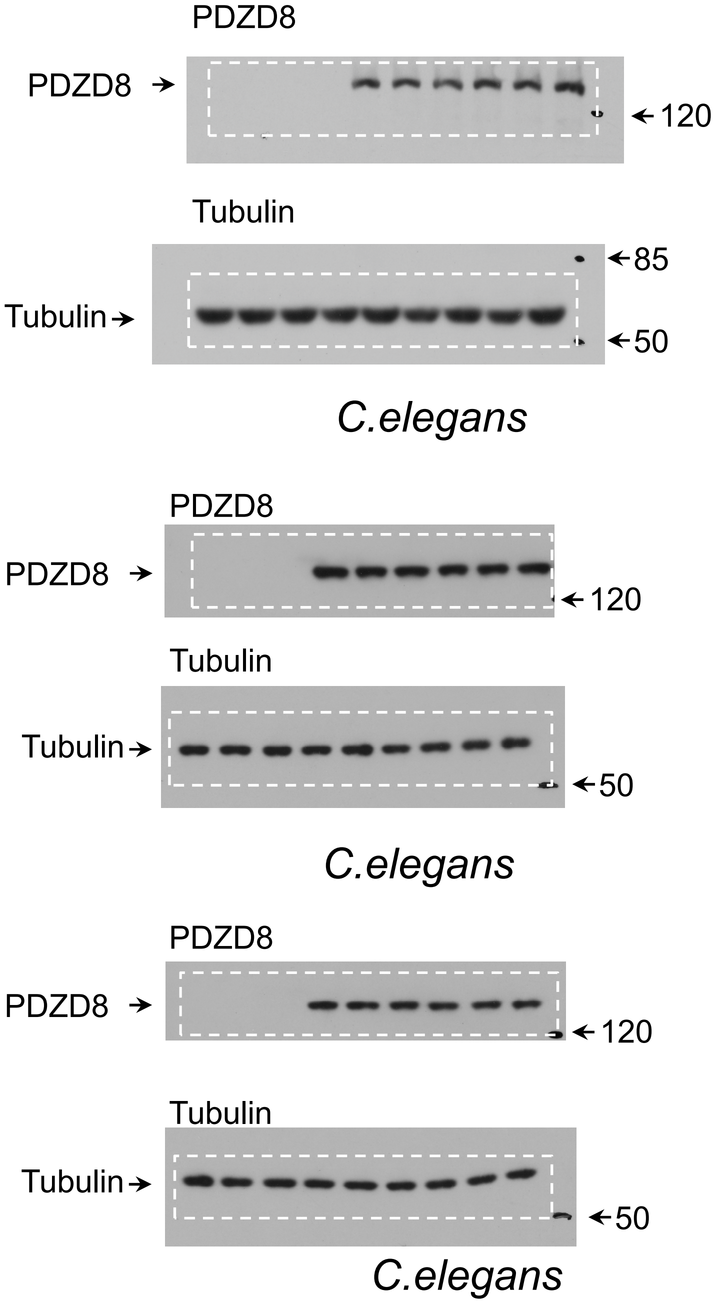

Fig. S2b

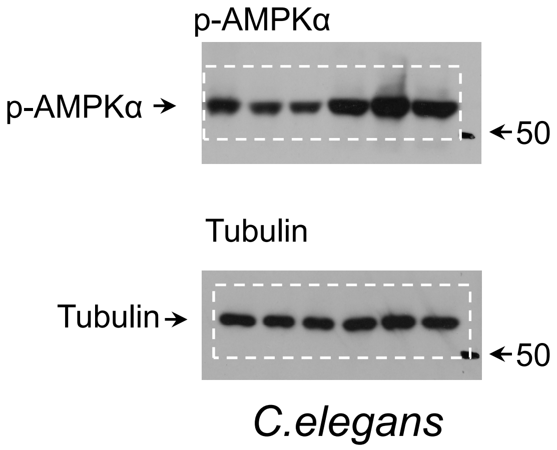

Fig. S4a

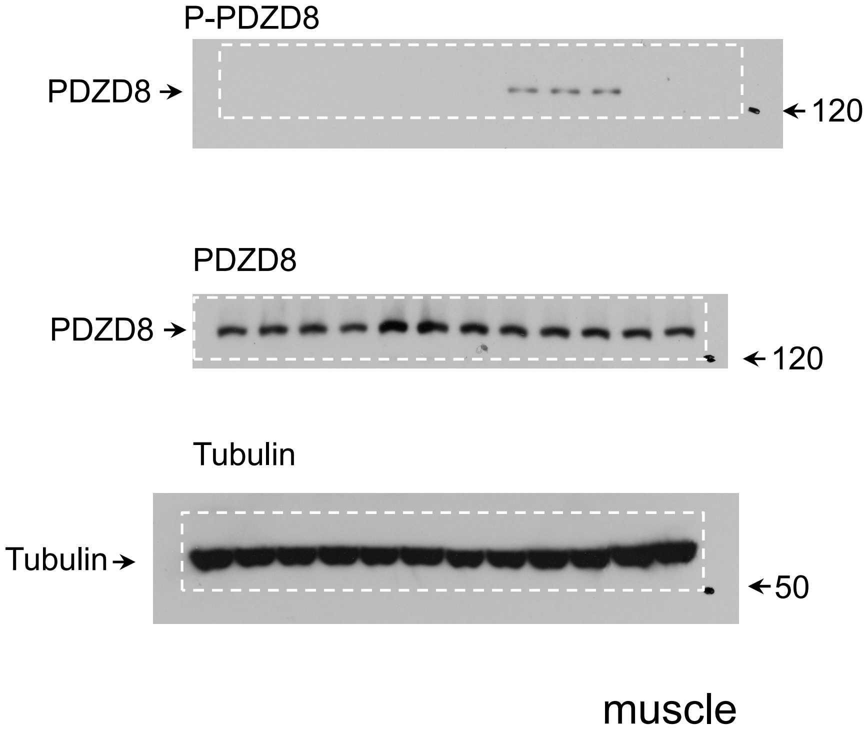

Supplement: Supplementary file 5 — Full scan [file 41422_2024_1021_MOESM5_ESM.pdf]
